# Effectiveness and safety of CD22 and CD19 dual‐targeting chimeric antigen receptor T‐cell therapy in patients with relapsed or refractory B‐cell malignancies: A meta‐analysis

**DOI:** 10.1002/cam4.6497

**Published:** 2023-09-05

**Authors:** Thi Thuy Nguyen, Nguyen Thanh Nhu, Chia‐Ling Chen, Chiou‐Feng Lin

**Affiliations:** ^1^ International Ph.D. Program in Medicine, College of Medicine Taipei Medical University Taipei Taiwan; ^2^ Department of Oncology Hue University of Medicine and Pharmacy, Hue University Hue Vietnam; ^3^ Department of Microbiology and Immunology, School of Medicine, College of Medicine Taipei Medical University Taipei Taiwan; ^4^ Faculty of Medicine Can Tho University of Medicine and Pharmacy Can Tho Vietnam; ^5^ School of Respiratory Therapy, College of Medicine Taipei Medical University Taipei Taiwan; ^6^ Core Laboratory of Immune Monitoring, Office of Research & Development Taipei Medical University Taipei Taiwan; ^7^ Graduate Institute of Medical Sciences, College of Medicine, Taipei Medical University Taipei Taiwan

**Keywords:** B cell malignancies, chimeric antigen receptor T (CAR‐T) therapy, dual‐targeting, efficacy, safety

## Abstract

**Background:**

The efficacy of CD22 or CD19 chimeric antigen receptor T (CAR‐T) cells in the management of acute lymphoblastic leukemia (ALL) and non‐Hodgkin lymphoma (NHL) was observed. Because antigen loss and lack of CAR‐T‐cell persistence are the leading causes of progressive disease following single‐antigen targeting, we evaluated CD22/CD19 dual‐targeting CAR‐T‐cell therapy efficacy and safety in relapsed/refractory B‐cell malignancies.

**Methods:**

The Web of Science, PubMed, Cochrane, and Embase databases were searched until July 2022. Patients confirmed with any relapsed/refractory B‐cell hematological malignancies were included regardless of age, gender, or ethnicity, receiving CD22 and CD19‐dual‐targeting CAR‐T‐cell therapy. The studies conducted on patients with coexisting other cancer were excluded. We used random‐effect models to explore the outcome, and heterogeneity was investigated by subgroup analysis.

**Results:**

Fourteen studies (405 patients) were included. The pooled overall response (OR) and complete remission (CR) were 97% and 93%, respectively, for ALL patients. The 1‐year proportions of overall survival (OS) and progression‐free survival (PFS) were 70% and 49%, respectively. For NHL, OR occurred in 85% of patients, and 57% experienced CR. The results illustrated that the 1‐year OS and 1‐year PFS were 77% and 65%, respectively. The subgroup analysis showed that the dual‐targeting modality achieved higher CR in the following cases: coadministration of CD22/CD19‐CAR‐T cells and third‐generation CAR‐T cells combined with ASCT and BEAM pretreatment. The ALL and NHL groups seemed similar in treatment‐related toxicity: all grade cytokine release syndrome (CRS), severe CRS, and neurotoxicity occurred in 86%, 7%, and 12% of patients, respectively.

**Conclusions:**

Our meta‐analysis demonstrated that the CD22/CD19 dual‐targeting CAR‐T‐cell strategy has high efficiency with tolerable adverse effects in B‐cell malignancies.

## Abstract 2

- This meta‐analysis demonstrates very high responses by using dual‐targeting CAR‐T therapy with a tolerable safety profile in treating R/R B‐cell hematologic malignancies.
- Although further studies are warranted, these findings may help immunologists and clinicians design proper CAR structure and/or combine with important target therapies to improve clinical outcomes.

## 1 INTRODUCTION

Among patients with relapsed and/or refractory (R/R) B‐cell malignancies, especially acute lymphoblastic leukemia (ALL) and non‐Hodgkin lymphoma (NHL), the impressive antitumor effects of chimeric antigen receptor T (CAR‐T) cell therapy have shown that it might be the most promising novel approach to treat such patients.^1^ CD19‐targeting CAR‐T‐cell therapy has recently achieved considerable efficacy in treating R/R ALL, with a remission incidence of 82%–93%.^2^, ^3^, ^4^ JULIET and ZUMA‐1 clinical trials have shown the significant clinical success of CAR‐T cells for treating R/R B‐cell NHL, and objective response rates have been observed at 52% and 82%.^5^, ^6^ However, despite the remarkable successes with CAR‐T cells in lymphoma and leukemia, 30%–50% of relapses after treatment are mainly characterized by CD19 antigen loss and lack of CAR‐T‐cell persistence.^7^, ^8^, ^9^ Similarly, a different target for CAR‐T cells is CD22 antigen, a molecule limited to the B‐cell lineage and expressed on most B‐cell malignancies. Currently, CD22 CAR‐T immunotherapy has achieved a 73% CR rate and median remission duration of 6 months with equal effectiveness, including in patients with previously infused CD19 CAR‐T cells and negative CD19‐antigen expression. However, reduced CD22‐antigen density at relapse is the main reason for treatment failure in patients receiving anti‐CD22 CAR‐T cell therapy, suggesting that CD22‐antigen escape through various mechanisms is possible.^10^, ^11^ Previous studies of treating solid tumors have demonstrated that dual‐antigen targeting may result in synergistic responses and prevent relapse compared with single‐antigen targeting,^12^, ^13^ which simultaneously targets CD22 and CD19 antigens. Dual‐targeting CAR‐T therapy can be produced in various approaches: coadministration of two different CAR‐T cell products, cotransduction of T cells with two different vectors, and a bicistronic vector encoding two CARs on the same T cells or a tandem CAR.^14^ The most interesting approach that is aiming at improving the efficacy of dual‐targeting CAR‐T cells is the infusion of a single T‐cell pool in which each T‐cell expresses two different CARs. The highly sensitive dual‐targeting co‐transduced CAR‐T‐cell therapy may be effective in preventing antigen escape.^15^, ^16^ Studies have reported CD19 CAR‐T pharmacokinetics in patients with ALL and NHL, allowing scientists to acknowledge the associations between the characterization of the cellular kinetics of CD19 CAR‐T and the efficacy or safety of these modalities.^17^, ^18^

Only two systematic reviews have documented the efficacy and safety of separated anti‐CD20 or CD19 CAR‐modified T cells for B‐cell malignancies but not dual‐targeting CD22/CD19‐CAR‐T‐cell therapy.^19^, ^20^ Additionally, the effects of a modality targeting a dual‐antigen CD22 and CD19, which demonstrate different mechanisms in persistence and behavior, remain unclear. The construct, product characteristics, and dose of infused dual‐targeting CAR‐T cells remain inconsistent. Hence, this study aimed to evaluate the clinical efficacy and safety of CD22/CD19 dual‐targeting CAR‐T‐cell therapy in R/R B‐cell malignancies.

## 2 METHODS

### 2.1 Registration and protocol

The systematic review protocol was registered on the “International Prospective Register of Systematic Review” under CRD42022329758. In addition, the review was performed and written as reported by the “Preferred Reporting Items for Systematic Reviews and Meta‐Analyses” (PRISMA) 2020 guideline.^21^

### 2.2 Eligibility criteria

#### 2.2.1 Study design

Prospective (all phases, controlled/ uncontrolled) and retrospective clinical trials and preliminary results of observational and interventional studies reported in full‐text articles were included. We included conference abstracts in a systematic review of qualitative analysis. Case series associated with a registered clinical trial were considered for inclusion. Letters, reviews, notes, case reports, comments, experiences that reported incomplete data, and cell or animal studies were excluded.

#### 2.2.2 Population

Patients confirmed with any R/R B‐cell hematological malignancies were included regardless of age, gender or ethnicity, receiving CD22 and CD19‐dual‐targeting CAR‐T‐cell therapy. The studies conducted on patients with coexisting other cancer were excluded.

#### 2.2.3 Intervention

While any B‐cell malignancy patients who received CD22/CD19 dual‐targeting CAR‐T‐cell therapy were considered, studies that used CD22/CD19 dual‐targeting CAR‐T‐cell therapy alone or combined with other treatments were included. Dual‐targeting CAR can be produced in different approaches: coadministration of CD22 CAR‐T cells and CD19 CAR‐T cell products, cotransduction of T cells with two vectors encoding the CD22 and CD19 CAR constructs, use of a bicistronic vector encoding both CD22 and CD19 CARs on the same T cells or use of a tandem CAR. Only second‐generation CARs and next‐generation CARs were included.^22^ Trivalent CAR‐T‐cell therapy was excluded.

#### 2.2.4 Outcomes

Response evaluations were determined according to “the US National Comprehensive Cancer Network guidelines and Lugano Treatment Response Criteria”.^23^ The primary outcomes were overall response (OR) and complete remission (CR). Secondary outcomes were partial response (PR). We defined the OR as the sum of CR and PR. For all studies, we recorded the percentage of patients who achieved a CR at 1 month and/or at 3 months, basing on the type of information reported. For all B‐ALL patients, CR was evaluated as the absence of detectable cancer, as <5% bone marrow blasts by morphology. A minimal residual disease (MRD)‐negative response was defined as a bone marrow blast proportion < 10^−4^ by multiparameter flow cytometry or real‐time quantitative polymerase chain reaction.^24^, ^25^ In hematological malignancies, partial response is considered when there has been a response to treatment but does not meet the criteria for CR. The additional secondary outcomes were overall survival (OS), and progression‐free survival (PFS). We defined OS as the day from the start of therapy to the day of death from any reason. PFS was defined the day from the start of therapy to day of disease progression. If available, OS and PFS rates were reported at 6 or 12 months. The toxicity results were classified into three main categories, cytokine release syndrome (CRS), severe CRS (sCRS), and neurotoxicity, by reporting the proportion of AEs. CRS was evaluated using the scale provided by Lee et al.^26^ or the Penn grading scale.^27^ sCRS was considered if it was Grade 3 or worse. Neurotoxicity, which was termed “immune effector cell‐associated neurotoxicity syndrome” (ICANS), and other AEs followed “the US National Cancer Institute Common Terminology Criteria for Adverse Events” (CTCAE v4.03 or v5.0).^26^, ^28^

Data sources, search strategies, and data extraction.

The PubMed, Web of Science, EMBASE, and Cochrane databases were searched with the terminological formula “chimeric antigen receptor” or “CAR T cells,” “CD19,” and “CD22” (Table S1). Briefly, titles and abstracts were reviewed to weed out duplicates and irrelevant studies on Endnote X9 (version 19; Clarivate. Philadelphia, PA, USA). Then, two independent evaluators conducted the study selection procedure and conferred with a third consultant when a dispute arose. If data were unavailable, we contacted the corresponding authors. For study features, we retrieved the first author's name, year of publication, sample size, patient characteristics, intervention parameters, outcomes, and AEs. We respectfully agreed with dividing ALL and NHL since the pathology and prognosis vary. Using the “methodological index for nonrandomized studies” (MINOR),^29^ the quality of the studies was evaluated.

### 2.3 Statistical analysis

Statistical analyses were performed using R software (version 4.0.2; R Foundation for Statistical Computing). Pooled effect sizes were computed using a random effect model. Subgroup analyses were conducted to explore the origin of heterogeneity. Hedges *Q* and *I* ^2^ statistics were used to estimate the quantity of heterogeneity among studies, with *I* ^2^ higher than 50% being considered as significant heterogeneity. *p* < 0.05 was considered as significant.

## 3 RESULTS

### 3.1 Literature search and study selection

**FIGURE 1:**
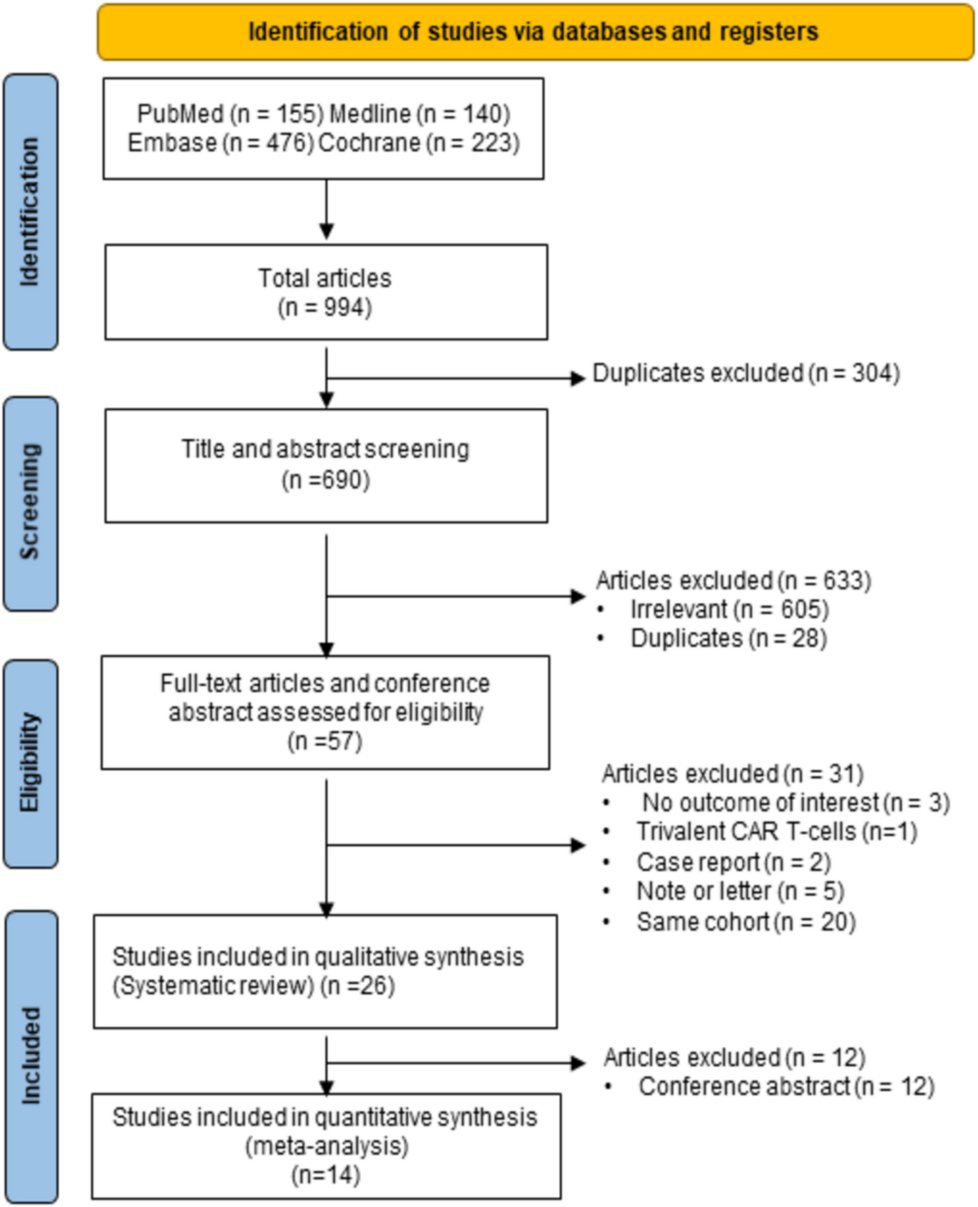
PRISMA flowchart summarizing the study selection process.

A total of 994 publications were collected from the databases through a systematic search in PubMed (*n* = 155), Embase (*n* = 476), Medline (*n* = 140), and the Cochrane Library (*n* = 223). Next, 690 articles were screened for their titles and abstracts. Of these publications, 633 were excluded due to irrelevancy (*n* = 605) and duplication (*n* = 28); thus, 57 articles remained for eligibility assessment. The other 31 papers were further eliminated because they reported trivalent CAR‐T cells (*n* = 1); involved case reports (*n* = 2), notes or letters (*n* = 5); did not include the outcome of interest (*n* = 3); or used the same sources of data to report (*n* = 20). Finally, 26 clinical trials met our inclusion criteria for a systematic review. Only 14 publications were pooled in the meta‐analysis because 12 articles described the patient baseline and outcomes through conference abstracts (Figure 1).

### 3.2 Study and participant characteristics

A total of twenty‐six articles were recruited from the studies. Most were prospective except for one retrospective study.^30^ Only one trial tested the bispecific CAR targeting CD22/CD19 in adults with both R/R ALL and NHL.^31^ All publications were published from 2019 to 2022 and were conducted in the USA, UK, and China. Only two studies reported the time between enrollment and CAR‐T‐cell infusion. Most trials demonstrated the number of enrolled patients that dropped out before receiving the CAR‐T‐cell infusion because of multiple reasons such as progressive disease, manufacturing failure, severe infection, death, etc. Among trials, only two patients in the Cao et al. study^32^ and one patient in Cordora et al. study^33^ were eliminated owing to difficulties in CAR‐T‐cell production (Table S2).

Four hundred five enrolled B‐cell malignancy patients (120 ALL and 285 NHL) were included in the quantitative analysis. Three trials had a sample size smaller than 10,^34^, ^35^, ^36^ and one study involved 123 patients.^37^ Fifty‐seven cases (47.5%) had one prior hematopoietic stem cell transplantation (HSCT), and the median follow‐up time was 12.8 months [4.3–19.7] (Table 1). For NHL studies, 11.9% of the patients had been pretreated with HSCT. The median follow‐up time was 14.1 months [8.7–24.3]. Eight NHL studies comprised 285 patients, of which 221 (77.5%) patients with diffuse large B‐cell lymphoma (DLBCL) were displayed (Table 2).

**TABLE 1:** CD22 and CD19 dual‐targeting chimeric antigen receptor T‐cell trials in patients with relapsed/refractory acute lymphoblastic leukemia.

ALL	PN	Patient age	Number of prior therapies	Prior HSCT	Dual CAR targeting	Infused CAR T‐cell dose	CAR construct and signaling/generation	Origin type of CAR T cells	T‐cell culture time (days)	Transduction method	Conditioning chemotherapy
Cordoba et al.^33^	15	8 (4–16)	2 (1–4)	7/15	Bicistronic vector	0.3 to 5 × 10^6^ CD19/22 CAR T cells/kg	scFv‐OX40‐TCRz (second generation) (anti‐CD19) and scFv‐41BB‐TCRz (second generation) (anti‐CD22)	Autologous	7–14	Retrovirus	Flu+Cyclo
Dai et al.^34^	6	28 (17–44)	2 (1–3)	0/6	Tandem	1.7 to 3 × 10^6^ CD19/22 CAR T cells/kg	scFv‐CD137‐CD3ζ (second generation) (anti‐CD22 and anti‐CD19)	Autologous	10	Lentivirus	Flu+Cyclo
Hu et al.^35^	6	49 (26–56)	5 (2–8)	1/6	Tandem	1 to 3 × 10^6^ CD19/22 CAR T cells/kg	scFv‐4‐1BB‐CD3ζ (second generation) (anti‐CD22 and anti‐CD19)	Allogeneic	11–13	Lentivirus	Flu+Cyclo+Alemtuzumab
Liu et al.^38^	21	21 (1.6–55)	>2	21/21	Coadministration	0.486 to 5.0 × 10^5^ CD19 CAR T cells/kg and 0.32–5.0 × 10^5^ CD22 CAR T cells/kg	scFv‐4‐1BB‐CD3ζ (second generation) (anti‐CD19) and scFv‐4‐1BB‐CD3ζ (second generation) (anti‐CD22)	Autologous	5–8	Lentivirus	Flu+/−Cyclo
Spiegel et al.^31^	17	47 (26–68)	>2	12/17	Tandem	1 to 3 × 10^6^ CD19/22 CAR T cells/kg	scFv‐4‐1BB‐CD3ζ (second generation) (anti‐CD22 and anti‐CD19)	Autologous	7–11	Lentivirus	Flu+Cyclo
Wang et al.^39^	51	27 (9–62)	2.5 (2–4)	12/51	Coadministration	2.6 ± 1.5 × 10^6^ CD19 CAR T cells/kg and 2.7 ± 1.2 × 10^6^ CD22 CAR T cells/kg	scFv‐CD28‐4‐1BB‐CD3ζ (third generation) (anti‐CD22) and scFv‐CD28‐4‐1BB‐CD3ζ (third generation) (anti‐CD19)	Autologous	12–13	Lentivirus	Flu+Cyclo
Zhang et al.^36^	4	28 (18–40)	3.5 (2–4)	4/4	Coadministration	1 × 10^6^ CD22 CAR‐T cells/kg and 1 × 10^6^/kg CD19 CAR‐T cells/kg	scFv‐4‐1BB‐CD3ζ (second generation) (anti‐CD22) and scFv‐4‐1BB‐CD3ζ (second generation) (anti‐CD19)	Allogeneic	10–15	Lentivirus	Flu+Cyclo

Abbreviations: Flu+Cyclo, fludarabine+cyclophosphamide; HSCT, hematopoietic stem cell transplantation; NR, not reported; PN, patient number.

**TABLE 2:** CD22 and CD19 dual‐targeting chimeric antigen receptor T‐cell trials in patients with relapsed/refractory non‐Hodgkin lymphoma.

NHL	PN	Disease type	Patient age	Number of prior therapies	Prior HSCT	Combined with CAR‐T‐cell therapy	Dual CAR targeting	Infused CAR T‐cell dose	CAR construct and signaling/generation	Origin type of CAR T cells	T‐cell culture time (days)	Transduction method	Conditioning chemotherapy
Cao et al.^32^	42	30 DLBC, 7 tFL, 2 DH HGBL, 3 others	41 (24–61)	4 (2–5)	0/42	HSCT	Coadministration	1.0 to 10.0 × 10^6^ CD22 CAR T cells/kg and 1.8 to 10.0 × 10^6^ CD19 CAR T cells/kg	scFv‐CD28‐4‐1BB‐CD3ζ (third generation) (anti‐CD22) and scFv‐CD28‐4‐1BB‐CD3ζ (third generation) (anti‐CD19)	Autologous	10–14	Lentivirus	BEAM protocol
Spiegel et al.^31^	21	14 DLBCL, 3 tFL, 2 Richter, 2 PMBCL	69 (25–78)	3.1 (2–7)	4/21	No	Tandem	1 to 3 × 10^6^ CD19/22 CAR T cells/kg	scFv‐4‐1BB‐CD3ζ (second generation) (anti‐CD22 and anti‐CD19)	Autologous	7–11	Lentivirus	Flu+Cyclo
Wei et al.^40^	16	10 DLBCL non‐GCB, 3 DLBCL GCB, 2 B‐LBL, 1 Burkitt lymphoma	52.5 (23–68)	2.9 (2–4)	1/16	No	Tandem	4.9–9.4 × 10^6^cells CD19/CD22 CAR T cells/kg	scFv‐4‐1BB‐CD3ζ (second generation) (anti‐CD22 and anti‐CD19)	Autologous	7–11	Lentivirus	Flu+Cyclo
Wei et al.^37^	123	86 DLBCL NOS, 17 DLBCL‐tFL, 10 HGBL DH/TH, 3 HGBL NOS, 4 Burkitt lymphoma, 1 MCL, 2 Others	44 (17–69)	3.2 (2–4)	14/123	HSCT in trial B	Coadministration	Trial A: 4.1 (1.4–8.9) × 10^6^ CD19 CAR T cells/kg and 6.0 (1.0–11.4) × 10^6^CD22 CAR T. Trial B: 4.0 (1.0–12.6) × 10^6^ CD19 CAR T cells/kg and 4.0 (0.8–10.0) × 10^6^ CD22 CAR T cells/kg	scFv‐CD28‐4‐1BB‐CD3ζ (third generation) (anti‐CD22) and scFv‐CD28‐4‐1BB‐CD3ζ (third generation) (anti‐CD19)	Autologous	12–13	Lentivirus	Flu+Cyclo or BEAM protocol
Wu et al.^43^	13	9 DLBCL non‐GCB, 3 DLBCL GCB, 1 ILBCL	42 (23–65)	3.1 (2–6)	0/13	HSCT	Coadministration	4.1 (2.6–8.4) × 10^6^ CD22 CAR T cells/kg and 4.3 (2.0–9.2) × 10^6^ for CD19 CAR T cells/kg	scFv‐CD28‐4‐1BB‐CD3ζ (third generation) (anti‐CD22) and scFv‐CD28‐4‐1BB‐CD3ζ (third generation) (anti‐CD19)	Autologous	12–13	Lentivirus	BEAM or Thiotepa‐based protocol
Zeng et al.^41^	14	12 DLBCL, 1 FL 3b, 1 MCL	47.5 (28–66)	4.5 (2–6)	3/14	No	Coadministration	5.6 (2.9–11.0) × 10^6^ CD22 CAR‐T cells/kg and 4 (2.1–8.0) × 10^6^ CD19 CAR‐T cells/kg	scFv‐CD28‐4‐1BB‐CD3ζ (third generation) (anti‐CD22) and scFv‐CD28‐4‐1BB‐CD3ζ (third generation) (anti‐CD19)	Autologous	10–14	Lentivirus	Flu+Cyclo
Zhang et al.^42^	32	17 DLBCL, 2 TFL, 1 PMBCL, 2 HGBL	<60: 24 ≥60: 8	>2	4/32	No	Tandem	3.69 × 10^8^ to 3.28 × 10^9^ CD19/22 CAR T cells	scFv‐4‐1BB‐CD3ζ (second generation) (anti‐CD22 and anti‐CD19)	Autologous	12	Lentivirus	Flu+Cyclo
Zhou et al.^30^	24	20 DLBCL, 2 TFL, 1 B‐LBL, 1 Burkitt lymphoma	51 (26–70)	2 (1–5)	8/24	No	NR	NR	NR	NR	NR	NR	NR

Abbreviations: B‐LBL, B‐cell lymphoblastic lymphoma; CNS, central nervous system; DH HGBL, double‐hit high‐grade B cell lymphoma; DLBCL NOS, diffuse large B‐cell lymphoma, not otherwise specified; DLBCL, diffuse large B‐cell lymphoma; DLBCL‐tFL, diffuse large B‐cell lymphoma transformed from FL; Flu+Cyclo, fludarabine+cyclophosphamide; GCB, germinal center B cell‐like; GCB, germinal center‐like B‐cell type; HGBL DH/TH, high‐grade B‐cell lymphoma with MYC and BCL2 and/or BCL6 rearrangements; HGBL NOS, HGBL not otherwise specified; HSCT, hematopoietic stem cell transplantation; ILBCL, intravascular large B‐cell lymphoma; MCL, mantle cell lymphoma; NR, not reported; PMBCL, primary mediastinal B cell lymphoma; PN, patient number; tFL, transformed follicular lymphoma.

Studies treating ALL patients were based on CD22/CD19 dual‐targeting CAR‐T‐cell treatment alone, including one study using bicistronic vector,^33^ two trials conducting cotransduction of CAR‐T cells, five studies infusing coadministration of CD22 and CD19 CAR‐T cell product^36^, ^38^, ^39^ and eight trials utilizing tandem CAR‐T cells.^31^, ^34^, ^35^ Most CAR‐T‐cell types originated from autologously transduced T cells, except for two studies using allogeneic T cells.^35^, ^36^ Most second‐generation CD19/CD22‐CARs were constructed, whereas Wang et al.^39^ used third‐generation products. T cells were transduced with retroviral or lentiviral vectors encoding anti‐CD22 and/or anti‐CD19 single‐chain variable fragments and were cultured for 5–15 days in vitro. Fludarabine and cyclophosphamide (FC) were the most commonly used lymphodepleting conditioning therapies. For NHL studies, dual‐targeting CAR‐T‐cell monotherapy or combined with other treatments were observed, of which only one study examined CAR22/19‐T‐cell therapy alone or in combination with HSCT in B‐cell malignancies.^37^ Dual CAR‐T‐cell therapy alone was given in six studies,^30^, ^31^, ^37^, ^40^, ^41^, ^42^ and a total of six studies administered dual‐targeting CAR‐T cells in combination with other treatments, including three trials that treated HSCT before or after CAR‐T‐cell infusion.^32^, ^37^, ^43^ All original T‐cell sources were autologous. Of note, all CAR‐T cells were in second‐generation^31^, ^40^, ^42^ or third‐generation^32^, ^37^, ^41^, ^43^ format. Before the 7–14 days of culture required to meet the dose, all T cells were transduced with a lentivirus encoding the CD22/CD19 target. Patients were pretreated with an FC‐based regimen or “Carmustine, Etoposide, Cytarabine, and Melphalan” (BEAM) preconditioning for lymphodepletion.

All studies used a scale developed by Lee and collaborators, except for the Shuangyou et al. trial,^38^ which used the Penn grading scale. ICANS and additional AEs were evaluated by “the American Society for Transplantation and Cellular Therapy” Consensus and CTCAE v4.03 or v5.0. (Table S3). For ALL, a total of seven studies with 120 patients who received CD22/CD19 CAR‐T cells were included in the meta‐analysis, in which 119 patients were eligible for the response evaluation, and 120 cases were used to assess the safety of dual‐targeting CAR‐T cells. The response was estimated at 28 days after CAR‐T infusion in five studies^31^, ^34^, ^35^, ^38^, ^39^; however, Cordoba et al.^33^ and Zhang et al.^36^ measured the response at 60 and 90 days. For NHL, eight articles with 285 patients were identified for eligibility in these trials, in which 281 patients were followed up to evaluate the response, and all 285 patients were included in the safety analysis. The response was measured for 1 month in four trials^30^, ^37^, ^40^, ^41^; however, in four trials,^31^, ^32^, ^42^, ^43^ the response was measured at 3 months.

### 3.3 Quality assessment

The mean MINORS score was 15 (range: 13–16), revealing considerable quality in the evidence base (Table S4).

### 3.4 Quantitative analysis

#### 3.4.1 Efficacy

**FIGURE 2:**
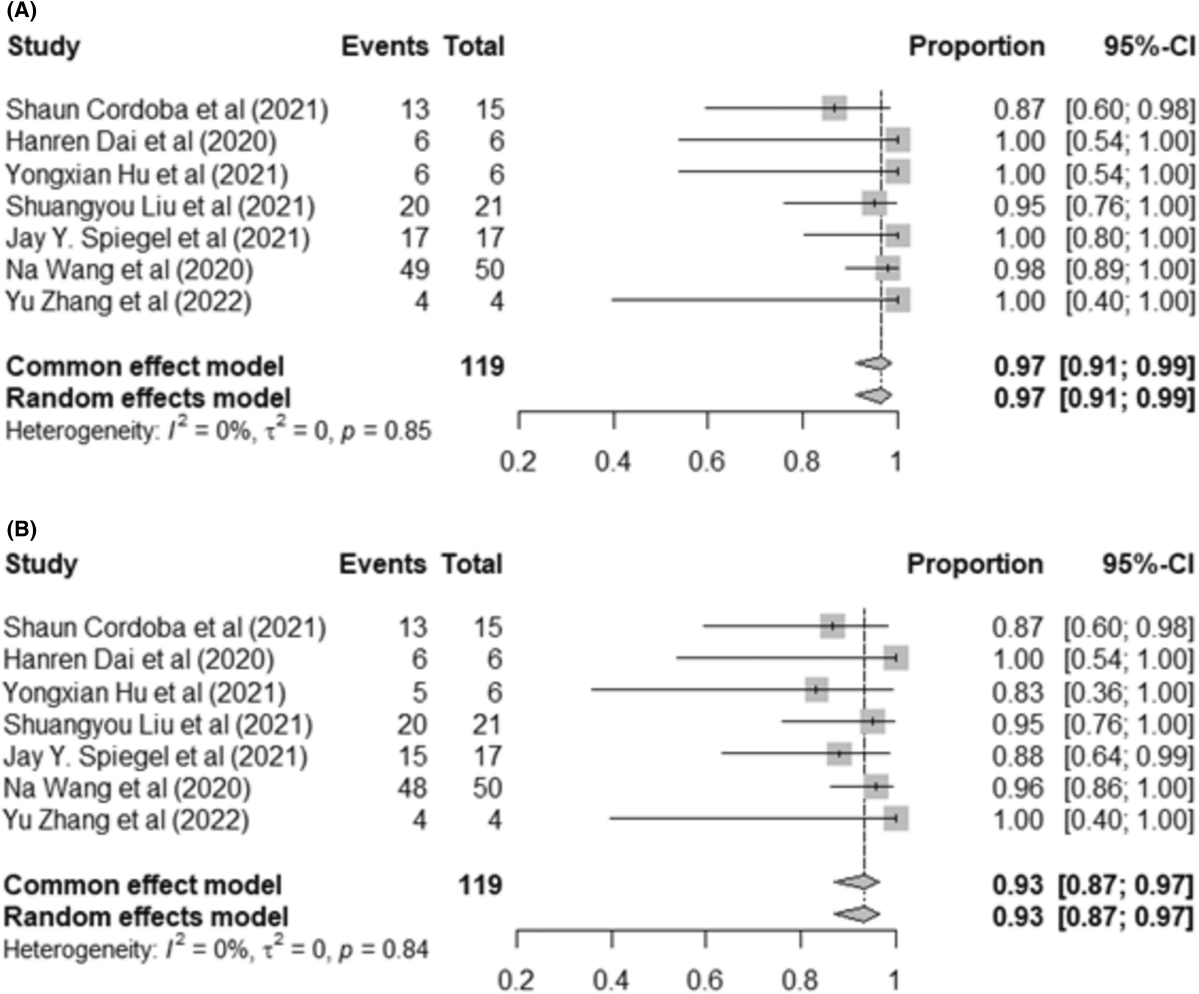
Forest plot showing the overall response and complete remission in relapsed/refractory ALL patients. (A) Overall response rates per phase I clinical trial. (B) Complete remission rates per phase I clinical trial. The study's sample size organizes forest plots within each first author and year of publication. Squares represent the estimated percentage, with the size of the squares representing the weight of each study. Horizontal lines crossing the squares express a 95% confidence interval (CI). The diamond represents the 95% CI of the pooled mean. The horizontal line at the lowest position demonstrates the prediction interval.

Among the seven included clinical trials in ALL, 119 patients were enrolled for the response rate evaluation. The pooled OR rate of CAR‐T‐cell therapy was 97% (95% CI: 91%–99%, *I* ^2^ = 0%). We observed that ALL patients who received dual target CAR T‐cell infusion had a high CR rate of 93% (95% CI: 87%–97%, *I* ^2^ = 0%) (Figure 2) and a low PR rate of 2% (95% CI: 0%–13%, *I* ^2^ = 0%) (Figure S1). The MRD‐negative remission rate was reported for 117 of the 120 enrolled patients. MRD‐negative CR was obtained in 109 out of 117 patients (93%, 95% CI: 87%–97%, *I* ^2^ = 0%) (Figure S2). The 6‐month survival analysis included 48 patients from five studies. The 6‐month OS and 6‐month PFS for this cohort were 83% (95% CI: 70%–91%, *I* ^2^ = 0%) and 50% (95% CI: 36%–64%, *I* ^2^ = 0%), respectively (Figure S3). Five studies had complete OS and PFS Kaplan–Meier curves at 12 months. The pooled 1‐year OS and 1‐year PFS were 70% (95% CI: 56%–80%, *I* ^2^ = 25%) and 49% (95% CI: 36%–62%, *I* ^2^ = 48%), respectively (Figure S4).

**FIGURE 3:**
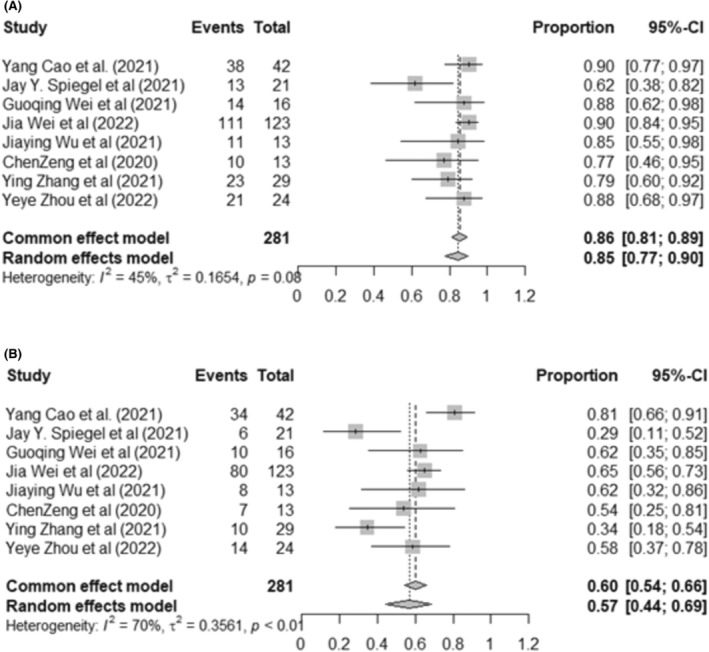
Forest plot showing the overall response and complete remission in relapsed/refractory NHL patients. (A) Overall response rates per phase I clinical trial. (B) Complete remission rates per phase I clinical trial. The study sample size organizes forest plots within each first author and year of publication. Squares represent the estimated percentage, with the size of the squares representing the weight of each study. Horizontal lines crossing the squares express a 95% confidence interval (CI). The diamond represents the 95% CI of the pooled mean. The horizontal line at the lowest position demonstrates the prediction interval.

For NHL, a total of 281 patients were identified for the tumor response in the eight NHL studies. The pooled OR rates reached 85% (95% CI: 77%–90%, *I* ^2^ = 45%). CR and PR were reported in eight trials, which were 57% (95% CI: 44%–69%, *I* ^2^ = 70%) (Figure 3) and 26% (95% CI: 19%–34%, *I* ^2^ = 37%), respectively (Figure S5). There was significant heterogeneity in the CR analysis. Therefore, subgroup analysis was performed for the dual‐targeting approach, CAR‐T generation, HSCT therapy combination, and lymphodepletion conditioning. We noted that patients who underwent infused coadministration of CD19‐CAR‐T cells and CD22‐CAR‐T cell products or third‐generation CAR‐T cells had a clinically significantly higher CR rate (68%, 95% CI: 59%–75%, *I* ^2^ = 39%) than patients who used tandem CAR or second‐generation CAR‐T cells (39%, 95% CI: 28%–52%, *I* ^2^ = 57%). All trials that combined HSCT treatment with dual‐targeting CAR‐T‐cell infusion achieved a higher CR rate (81%, 95% CI: 73–87, *I* ^2^ = 48%) than those that used dual‐targeting CAR‐T therapy or FC for lymphodepletion (46%, 95% CI: 39–54, *I* ^2^ = 33%) (*p* < 0.01). All studies that used the BEAM regimen during lymphodepletion achieved a higher CR rate (85%, 95%: 76–90, *I* ^2^ = 0%) than those that treated FC for lymphodepletion (46%, 95% CI: 39–53, *I* ^2^ = 22%) (*p* < 0.01) (Figure S6). A total of 145 patients from six clinical trials were included for the 12‐month survival analysis. The 12‐month OS and 12‐month PFS were 77% (95% CI: 65%–85%, *I* ^2^ = 50%) and 56% (95% CI: 36%–75%, *I* ^2^ = 80%), respectively (Figure S7).

### 3.5 Safety

**FIGURE 4:**
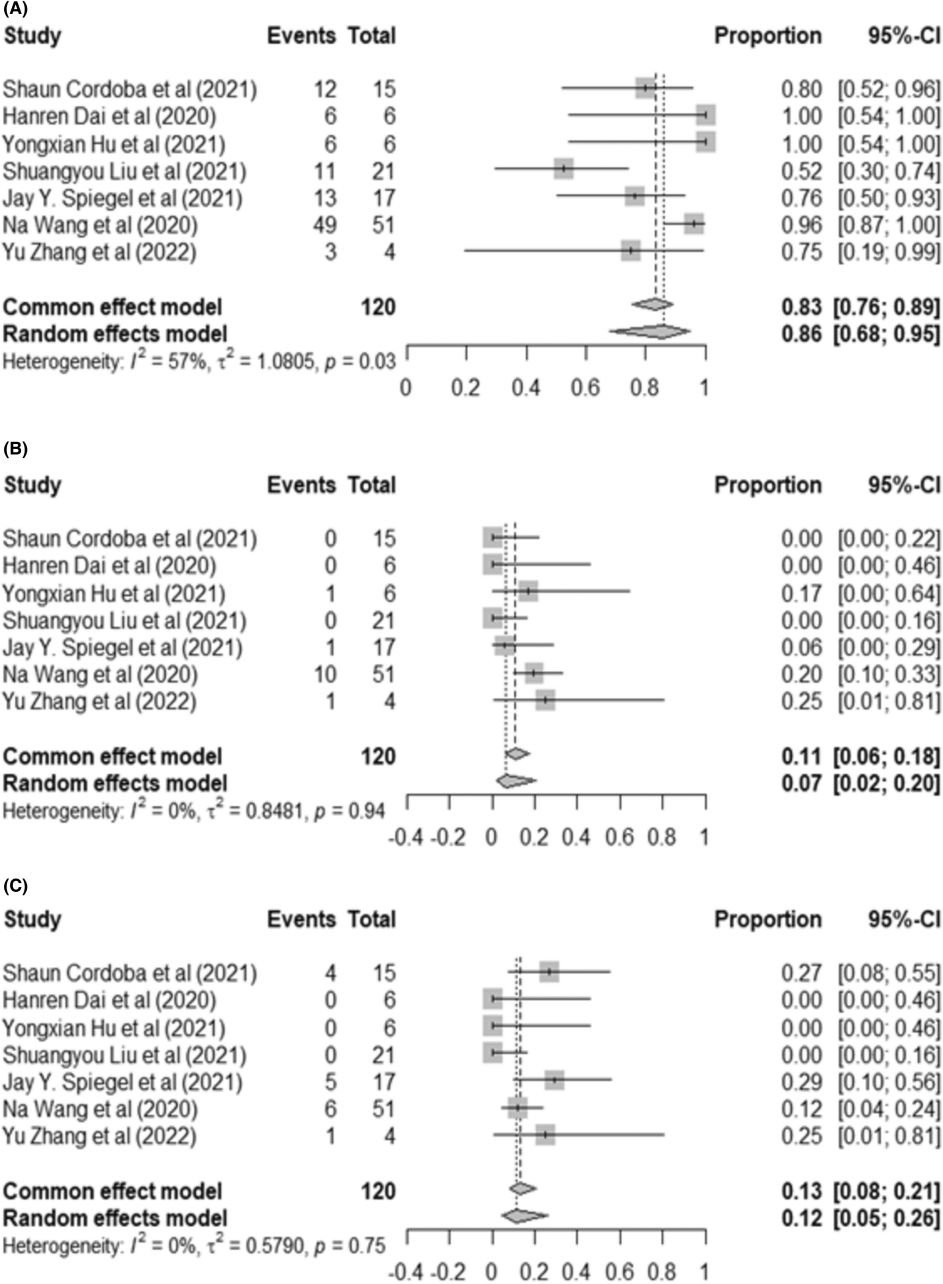
Forest plot of the most common adverse events in relapsed/refractory ALL patients. (A) Cytokine release syndrome. (B) Severe cytokine release syndrome. (C) Neurotoxicity.

For ALL, data for safety were available for 120 patients. We observed that CRS events were 86% (95% CI: 68%–95%, *I* ^2^ = 57%), 7% of which developed sCRS with 95% CI: 2%–20%, *I* ^2^ = 0%. Neurotoxicity was described for 120 cases, with 12% (16 cases) with 95% CI: 5%–26%, *I* ^2^ = 0% (Figure 4). The studies using the Lee et al. scale resulted in a pooled CRS of 90% (95% CI: 79%–95%) with low heterogeneity (*I* ^2^ = 12%, *p* = 0.11). While the second‐generation dual‐targeting CAR‐T therapy showed a lower pooled CRS of 78% (95% CI: 59%–89%, *I* ^2^ = 35%), the third‐generation CAR‐T‐cell therapy reported a pooled CRS of up to 96% (95% CI: 87%–100%). The CRS in the two subgroups was significantly different, with *p* = 0.04. In the subsequent subgroup analyses, we also demonstrated that clinical trials that infused a high number of CAR‐T cells had a higher percentage of CRS (90%, 95% CI: 80%–95%, *I* ^2^ = 12%) than trials that used a lower dose of CAR‐T cells (52%, 95% CI: 30%–74%) (*p* < 0.01) (Figure S8).

**FIGURE 5:**
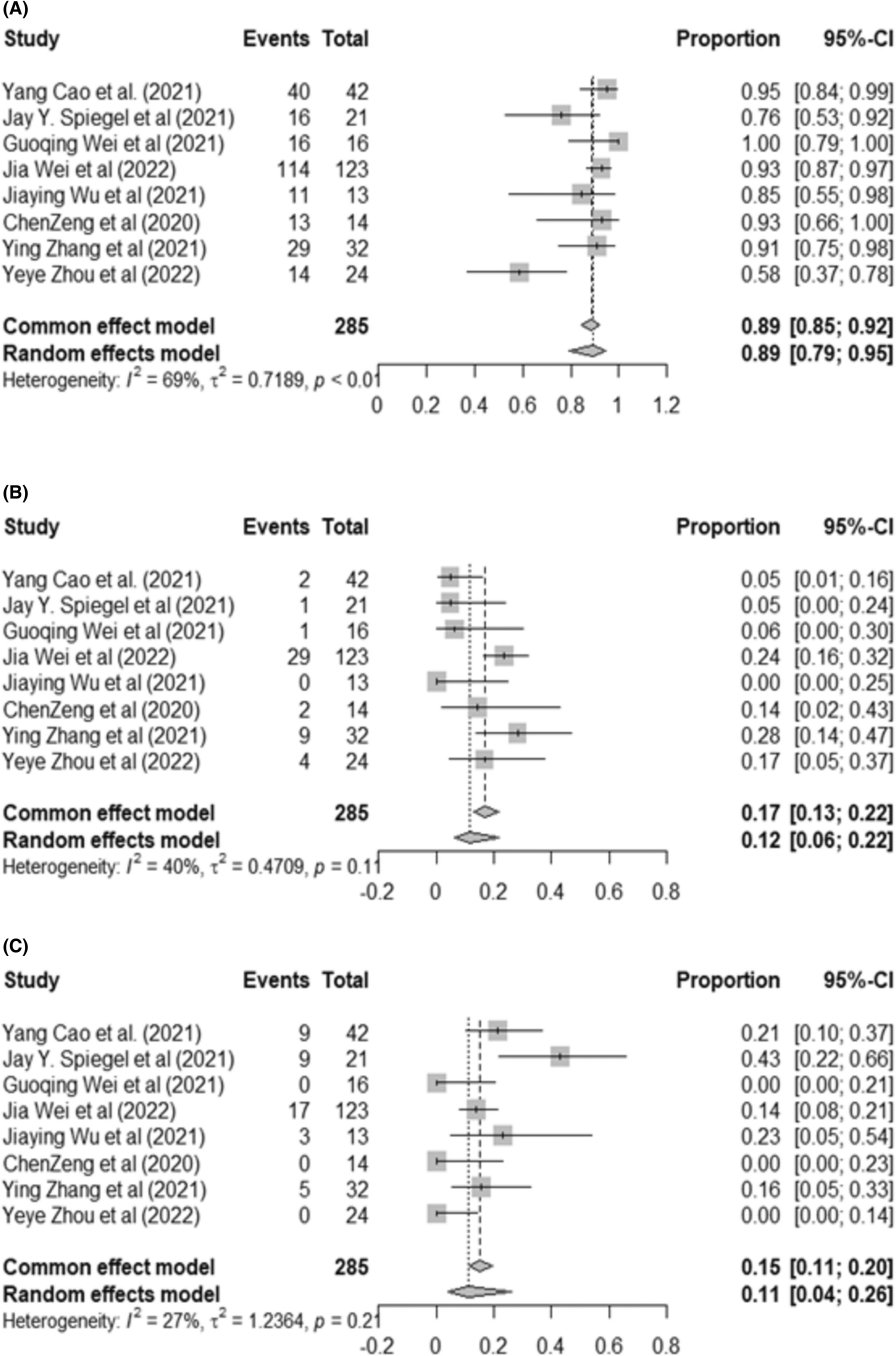
Forest plot for the most common adverse events in relapsed/refractory NHL patients. (A) Cytokine release syndrome. (B) Severe cytokine release syndrome. (C) Neurotoxicity.

For NHL, 89% (95% CI: 79%–95%, *I* ^2^ = 69%) of patients experienced CRS of any grade, whereas 12% (95% CI: 6%–22%, *I* ^2^ = 40%) of cases developed sCRS. In addition, ICANS occurred in 11% (95% CI: 4%–26%, *I* ^2^ = 27%) of patients during the dual‐targeting CAR‐T‐cell treatment (Figure 5). Significant heterogeneity in CRS was observed among studies. In the following subgroup analysis, we found that trials that used third‐generation CAR‐T cells or coadministration dual‐targeting CAR‐T therapy had a significantly higher rate of CRS (93%, 95% CI: 88%–96%, *I* ^2^ = 0%) than studies that used second‐generation CAR‐T cells or tandem anti‐CD22‐CD19 constructs (88%, 95% CI: 78%–94%, *I* ^2^ = 0%) (*p* < 0.01). The pooled CRS in the two groups was significantly different, with *p* < 0.01 (Figure S9).

### 3.6 Publication bias

Publication bias was assessed using a funnel plot and Egger's test. Assessment results of the pooled OR, CR, CRS, and neurotoxicity did not identify significant publication bias among studies, with *p* > 0.05, except for sCRS in NHL studies (*p* = 0.007) (Table 3) (Figure S10).

**TABLE 3:** Analysis of publication bias by Egger's test.

Outcome		Intercept	SE	Slope	*t*‐Test	df	*p*‐value
ALL	OR	0.79	1.11	1.84	0.72	5	0.50
	CR	−0.22	1.06	2.57	−0.21	5	0.84
	CRS	2.32	1.36	−0.32	1.7	5	0.14
	sCRS	−1.22	0.50	−0.94	−2.43	5	0.06
	Neurotoxicity	−0.92	0.91	−0.98	−1.01	5	0.36
NHL	OR	−0.91	1.65	2.13	−0.56	6	0.59
	CR	−1.47	1.67	0.89	−0.88	6	0.41
	CRS	1.28	1.76	1.14	0.73	6	0.49
	sCRS	−1.95	0.49	−0.65	−3.93	6	0.007
	Neurotoxicity	−1.05	1.06	−1.07	−0.98	6	0.36

Abbreviations: CR, complete remission; CRS, cytokine release syndrome; df, degree of freedom; OR, overall response; sCRS, severe cytokine release syndrome; SE, standard error.

## 4 DISCUSSION

### 4.1 Summary of main findings

Our study is the first to evaluate the effectiveness and safety of CD22/CD19 dual‐targeting CAR‐T‐cell therapy in patients with R/R B‐cell malignancies. Our findings suggested a range of 5 × 10^6^ cells/kg CAR‐T cells or higher, autologous CAR‐T cells, FC or BEAM regimens, and viral transduction as the recommended methods. Our findings were summarized as follows: (1) ALL patients treated with dual‐targeting CAR‐T cells demonstrated a considerably better OR and CR rate of 97% and 93%, respectively; (2) the best OR of dual‐targeting CAR‐T‐cell therapy for NHL was as high as 85%, and the CR was 57%; and (3) CRS and neurotoxicity are significant side effects in dual‐targeting CAR‐T‐cell therapy. Although 86% of patients experienced CRS of any grade after dual‐targeting CAR‐T‐cell infusion, sCRS was found in 7% of ALL cases. For NHL, grade ≥3 CRS was observed in 12% of patients, while 89% developed CRS of any grade. Only 12% of ALL or NHL patients suffered any grade of neurotoxicity. (4) Dual‐targeting CAR‐T cells showed a significantly higher response among ALL patients than among NHL patients. The current review suggested that the efficacy of CD22/CD19 dual‐targeting CAR‐T‐cell therapy is superior to that of single‐targeting CAR‐T cells such as CD19 or CD22 antigen alone, with a tolerable safety profile in treating R/R B‐cell malignancies.

Clinical trials of CAR‐T‐cell therapy often show better results than real‐world experiences with these therapies. One of the reasons for this difference is that patients enrolled in these trials must endure a considerable wait time between deciding to undergo CAR‐T therapy and receiving the infusion. As a result, the selection bias favors patients with less aggressive diseases and those who can tolerate bridging therapy.^44^, ^45^ Most of the studies in this meta‐analysis did not report the time between trial enrollment and CAR‐T infusion, but the T‐cell culture time ranged from 5 to 15 days. Waiting for CAR‐T cells to be manufactured could be longer than this duration. The number of enrolled patients that dropped out before being able to receive the CAR‐T infusion could be acceptable in patients with relapsed/refractory B‐cell malignancies. In addition, these trials were conducted in the multicenter US, UK, and China, so the rate of CAR‐T production failure was slightly low.

Similar to previous studies,^19^, ^20^ we identified that this method could enhance the response in ALL and NHL patients, suggesting that dual‐targeting CAR‐T‐cell therapy could exhibit superior anti‐B‐cell malignancy efficacy. In line with this theory, the meta‐analysis of Aamir et al.^46^ reported that the CR was 82% in ALL patients. Consistently, other meta‐analyses showed that anti‐CD19 CAR‐T therapy achieved a high mean CR rate (81%) at 4 weeks.^47^, ^48^ Our study demonstrated that CD22/CD19 dual‐targeting CAR‐T‐cell therapy could tremendously alleviate symptoms in 97% of patients, confirming previous publications highlighting CAR‐T‐cell therapy's benefits in ALL patients. The estimated 1‐year PFS and OS in the current report of 49% and 70% appear higher than those reported in other cohorts, with PFS and OS inferior to 37% and 58%.^47^, ^48^ The results presented in this meta‐analysis do not directly compare to the randomized clinical trial results of single‐targeting CAR‐T cells or other targeted immunotherapies but allow basic estimates for consideration. Furthermore, the recent data of dual‐targeting CAR‐T‐cell therapy reported in the conference abstract are shown in Table S5, which might further confirm the safety and effectiveness in ALL patients.

A systematic review and meta‐analysis in which CD19‐CAR‐T cells were administered to 280 NHL adults reached a total OR and CR of 63% and 33%, respectively.^49^ Although no study compared dual‐targeting and single‐targeting CAR‐T‐cell therapy, dual‐targeting CAR‐T cells appeared to have considerable advances compared to traditional CAR‐T cells. The current analysis showed a promising outcome with a 1‐year PFS of 56%, demonstrating a trend toward improved survival, while other CAR‐T cells remained at a low 1‐year PFS of 48%.^20^ Even though the available data are limited, our subgroup analysis illustrated that third‐generation CAR‐T cells reported higher responses than second‐generation CAR‐T cells (*p* < 0.01). These correlations can be clarified by clinical information, which suggests that 4‐1BB costimulatory domains are needed for the persistence of CAR‐T cells, while the CD28 signaling domain leads to a quick response but restricted perseverance.^50^, ^51^ Zhou et al.^20^ discovered that the 4‐1BB signaling domain was associated with lower survival compared to CD28 domains (*p* = 0.0489). BEAM is the most commonly applied conditioning regimen for NHL.^52^ In our analysis, patients who received a standard BEAM regimen before infusion of CAR‐T cells had a higher CR, and the potential superiority of BEAM over FC conditioning in leading to positive outcomes needs to be addressed in further studies. Combining autologous stem cell transplantation (ASCT) and CAR‐T‐cell approaches has shown superior antilymphoma efficacy and safety.^53^, ^54^ Herein, we report the clinical effectiveness of dual‐targeting CAR‐T cells and ASCT in R/R NHL patients. Despite the absence of a head‐to‐head clinical trial, the CR of the combined CAR‐T‐cell and ASCT approach relatively surpassed those of another independent research group with dual‐targeting therapy alone in NHL patients without a difference in toxicities. Although the fundamental mechanisms of the combination treatment are still unclear, the above results demonstrate that ASCT could have a potential synergistic antilymphoma effect.

We divided our sample into two cohorts to minimize the significant source of heterogeneity. We found that the efficacy was significantly higher for ALL patients than for NHL patients. However, we did not perform a comparative statistical calculation. Prior studies proposed that CAR‐T treatment still has many challenges for B‐cell lymphoma, such as antigen loss and the expression of immune checkpoint molecules.^5^, ^55^ These problems led to a poorer response rate of NHL cases, and our current results further validate this finding. Because NHL has features intermediate between solid tumors and leukemia, a more crucial immunosuppressive microenvironment in lymphoma would restrict the antitumor function of CAR‐T cells. To overcome the aggressive tumor microenvironment and reduce suppression of effector cells, intensification of lymphodepletion must be infused before or after CAR‐T‐cell infusion. Thus, multiple levels of lymphoproliferative cytokines may be allowed to maximize the function of adoptive T cells^52^ successfully. Based on the above hypothesis, two authors recently conducted clinical trials of dual‐targeting CAR‐T cells combined with anti‐programmed death‐1 (anti‐PD‐1) antibody in R/R DLBCL and reported promising outcomes with reasonable AEs. In addition, further modification of the CAR structure and an ideal strategy to treat the patients who fail to receive autologous CAR‐T cells are needed to use haploidentical HSCT with conditioning, including allogeneic CAR‐T cells targeting both CD22 and CD19 from the same donor. We summarized the recent study characteristics of dual‐targeting CAR‐T‐cell therapy reported in the conference abstract (Table S6). This result indicated that the dual‐targeting CAR‐T‐cell effectiveness and toxicity profile in R/R B‐NHL were improved when combined with other new treatments.

For ALL patients, the safety profile illustrated a high rate of CRS but a low percentage of Grade 3 or worse CRS and neurotoxicity. Previous studies have shown a similar trend related to CRS but a higher sCRS rate: 82% of patients developed CRS of any grade, and 27% of patients developed grade 3 or higher CRS. Likewise, these studies reported a higher neurotoxicity percentage (29%–34%) after anti‐CD19 CAR‐T‐cell infusion.^47^, ^48^ Although comparison with anti‐CD19 CAR‐T infusion is limited by various CAR constructs and signaling, these results at least support that dual‐targeting CAR‐T‐associated toxicity is tolerable in B‐ALL. This information has shown that the safety of CAR‐T cells in B‐ALL in children and/or adults was improved. The current meta‐analysis demonstrated a similar tendency for ALL patients to be as safe as NHL patients. Notably, the actual toxicity among all R/R NHL patients who were infused with the second‐generation CAR‐T cells or tandem anti‐CD22‐CD19 construct might be lower. A previous study reported that a higher rate of patients with DLBCL treated with CD28 costimulatory domain anti‐CD19 CAR‐T cells had CRS than those treated with 4‐1BB costimulation anti‐CD19 CAR‐T cells.^56^

### 4.2 Limitations

Our study has several limitations. First, we included relatively small studies and single‐arm clinical trials from a single institution due to the wide clinical utilization of CAR‐T‐cell therapy. No randomized controlled trial was performed on the subject of interest at the time of the analysis. Thus, any conclusions might lead to a high risk of interpretation bias. However, we enrolled the inclusion and exclusion criteria from different publications of acceptable quality, allowing our meta‐analysis to have high internal validity. Notably, the subgroup analysis results reduced the heterogeneity and would have been informative due to the individual heterogeneity and the different sample sizes. We also examined the clinical trials in conference abstracts to guarantee the controlled variables and the accuracy of the final results. Second, because the follow‐up durations were short and the outcome window was highly variable between studies, the conclusion on the durability of the response to CAR‐T‐cell therapy was limited. Third, we were also unable to exclude some clinical parameters, such as age, specific disease indication, different CAR‐T constructs, the variability in the use of chemotherapy, dose, nature of lymphodepletion, and various inclusion and exclusion criteria, through studies. Fourth, we used the response rate for efficacy estimates as the included studies most commonly reported. However, the response rate is a surrogate outcome, which may not correlate with prolonged survival or improved patient outcomes.^57^ It was not practicable to conduct such analysis because of limited data from original studies, even after we contacted the authors, because of the lack of consistent time reporting of each study. Because all studies are still in phase I, we could not perform a functional analysis of OS and PFS. Fifth, we observed statistical proof of publication bias regarding sCRS in R/R NHL studies. However, in this meta‐analysis, we applied a careful study methodology for reliability. Sixth, the duration or degree of CAR‐T cell expansion and persistence and B‐cell aplasia were not well described across studies due to limited sources. Finally, we perceive that despite the efforts of two authors to screen all literature on this topic, our search strategy might have missed some studies and the most recent updates, given the quickly advancing modality in this field.

### 4.3 Implement and perspective

These results can significantly influence the management of R/R B‐cell malignancies. In addition, these findings can be helpful for immunologists and clinicians in terms of designing proper CAR structure and signaling or generation, techniques to establish CARs into T cells, CAR‐T‐cell dosages, origin type of CAR‐T cells (autologous or allogeneic), T‐cell culture time and conditions, lymphodepletion condition, cell subtypes used to initiate CAR‐T cells and cytokine administration.

To increase the efficacy and safety of available CAR‐T‐cell therapy, new strategies should be considered related to CAR products, combined treatment, patient selection, and specific lymphodepletion. Further multi‐institution phase II prospective clinical trials are needed to establish CD22/CD19 dual‐targeting CAR‐T‐cell therapy as a standard modality for R/R ALL and NHL patients. A direct comparison of the effectiveness and safety of CD19 targeting or CD22 targeting versus CD22/CD19 dual‐targeting CAR‐T cells is absent. Questions of the superiority of single‐targeting over dual‐targeting need to be elucidated in prospective studies. There are currently no direct comparison studies between different anti‐CD22 and anti‐CD19 CAR‐T‐cell approaches, such as cocktail/sequential administration, cotransduction, bicistronic or tandem modality, and conclusions regarding their efficacy and toxicity can only be drawn from meta‐analyses and mechanistic studies. There is an urgent need for well‐designed randomized controlled trials that compare different dual‐targeting CAR‐T‐cell therapies, dual‐targeting therapy plus autologous or allogeneic HSCT, or dual‐targeting therapy combined with important immune checkpoint inhibitors versus dual‐targeting therapy alone. Future work is essential to optimize dual‐targeting or multitarget CAR‐T cells to enhance the effectiveness of these modalities in B‐cell malignancies and solid tumors.

## 5 CONCLUSION

Our study demonstrates very high responses by using dual‐targeting CAR‐T therapy in R/R B‐cell malignancies and proposes a benchmark for the improvement of CAR‐T‐cell therapy. Despite the limitation due to phase I, single‐arm clinical trials and the heterogeneity of reported studies, the results suggest that dual‐targeted CAR‐T therapy should be considered for cancer patients and that clinicians should take care of cancer patients to further advance improvement in this domain. However, further alterations of CAR structure and ideal therapy modality are required in ongoing or further clinical trials to obtain adequate treatment for cancer patients.

## AUTHOR CONTRIBUTIONS

**Thi Thuy Nguyen:** Conceptualization (lead); data curation (equal); formal analysis (lead); funding acquisition (supporting); investigation (equal); methodology (equal); project administration (supporting); resources (supporting); software (equal); validation (equal); visualization (equal); writing – original draft (lead); writing – review and editing (lead). **Nguyen Thanh Nhu:** Data curation (equal); formal analysis (equal); investigation (equal); methodology (equal); project administration (supporting); resources (supporting); software (equal); validation (equal); visualization (equal). **Chia‐Ling Chen:** Data curation (supporting); supervision (supporting); validation (supporting); writing – review and editing (supporting). **Chiou‐Feng Lin:** Conceptualization (supporting); data curation (supporting); formal analysis (supporting); funding acquisition (lead); investigation (supporting); methodology (supporting); project administration (lead); resources (lead); software (supporting); supervision (lead); validation (supporting); visualization (supporting); writing – review and editing (lead).

## FUNDING STATEMENT

This work was supported by grants (MOST110‐2320‐B038‐064‐MY3, MOST111‐2327‐B006‐005, MOST111‐2320‐B038‐012, and MOST111‐2314‐B038‐120‐MY3) of the National Science and Technology Council in Taiwan and the industry‐academia cooperation program (TMU‐MSD A‐111‐112).

## CONFLICT OF INTEREST STATEMENT

The authors declare without conflicts of interest.

## Supporting information


**Data S1.** Supporting Information

## Data Availability

Data were extracted and analyzed from published articles, all available and accessible in the common database. All datasets generated during the study are available upon reasonable request from the corresponding authors. The study protocol has been published (ID: CRD42022329758; www.crd.york.ac.uk/PROSPERO/) and is universally available.
