# Supplementary material for: Effectiveness and safety of CD22 and CD19 dual‐targeting chimeric antigen receptor T‐cell therapy in patients with relapsed or refractory B‐cell malignancies: A meta‐analysis
Source: Cancer Med. 2023 Sep 5;12(18):18767–85. doi: 10.1002/cam4.6497 (PMC10557829; doi:10.1002/cam4.6497)

**SUPPLEMENTARY APPENDIX**

**Table A.1.** **Search strategy (all fields included)**

| **PubMed** | | |
| --- | --- | --- |
| **No** | **Query** | **Results** |
| #1 | "receptors, chimeric antigen"[MeSH Terms] OR ("receptors"[All Fields] AND "chimeric"[All Fields] AND "antigen"[All Fields]) OR "chimeric antigen receptors"[All Fields] OR ("chimeric"[All Fields] AND "antigen"[All Fields] AND "receptor"[All Fields]) OR "chimeric antigen receptor"[All Fields] | 9,384 |
| #2 | "CD19"[All Fields] | 13,914 |
| #3 | "CD22"[All Fields] | 2,481 |
| #4 | #1 AND #2 AND #3 | 155 |
| **EMBASE** | | |
| #1 | 'chimeric antigen receptor'/exp OR 'chimeric antigen receptor' | 18,989 |
| #2 | 'cd19 antigen' | 21,723 |
| #3 | 'cd22 antigen' | 3,881 |
| #4 | #1 AND #2 AND #3 | 476 |
| **MEDLINE** | | |
| #1 | chimeric antigen receptor.mp. or exp Receptors, Chimeric Antigen/ | 6,949 |
| #2 | CD19.mp. | 13,765 |
| #3 | CD22.mp. | 2,445 |
| #4 | #1 AND #2 AND #3 | 140 |
| **Cochrane library** | | |
| #1 | CAR T cells | 444 |
| #2 | chimeric antigen receptor | 246 |
| #3 | CD19 | 963 |
| #4 | CD22 | 157 |
| #5 | #1 OR #2 AND #3 AND #4 in Trials | 223 |

**Table A.2.** **Patient enrollment and baseline features**

| **Study** | **The time between study enrollment and CAR T infusion** | **Number of enrolled patients** | **Number of patients receiving CAR T infusion** | **Number of enrolled patients that dropped out before receiving CAR T infusion** | **Number of patients with CAR T production failure** |
| --- | --- | --- | --- | --- | --- |
| Yang Cao et al. | NR | 49 | 42 | 7 | 2 |
| Shaun Cordora et al. | NR | 20 | 15 | 5 | 1 |
| Hanren Dai et al. | NR | 6 | 6 | 0 | 0 |
| Yongxian Hu et al. | ≤ 8 days | 6 | 6 | 0 | 0 |
| Shuangyou Liu et al. | NR | 32 | 21 | 11 | 0 |
| Jay Y. Spiegel et al. | ≥ 14 days | 39 | 38 | 1 | 0 |
| Na Wang et al. | NR | NR | 51 | NR | 0 |
| Guoqing Wei et al. | NR | 18 | 16 | 2 | 0 |
| Jia Wei et al. | NR | NR | NR | NR | NR |
| Jiaying Wu et al. | NR | 13 | 13 | 0 | 0 |
| ChenZeng et al. | NR | 14 | 14 | 0 | 0 |
| Ying Zhang et al. | NR | 34 | 32 | 2 | 0 |
| Yu Zhang et al. | NR | 4 | 4 | 0 | 0 |
| Yeye Zhou et al. | NR | 24 | 24 | 0 | 0 |

Abbreviations: NR, no reported.

**Table A.3.** **Toxicity and Response Assessment**

| **Study** | **CRS** | **Neurotoxicity**  **(ICANS)** | **Response** |
| --- | --- | --- | --- |
| Yang Cao et al. | Lee DW et al | CTCAE v4.03 | NCCN guidelines and Lugano Treatment Response Criteria |
| Shaun Cordora et al. | Lee DW et al | CTCAE v5.0 | NCCN guidelines |
| Hanren Dai et al. | Lee DW et al | CTCAE v5.0 | NR |
| Yongxian Hu et al. | Lee DW et al | NR | NR |
| Shuangyou Liu et al. | Penn grading scale | CTCAE v5.0 | NCCN guidelines |
| Jay Y. Spiegel et al. | Lee DW et al | NR | Lugano PET/CT criteria |
| Na Wang et al. | Lee DW et al | CTCAE v4.03 | NCCN guidelines and Lugano Treatment Response Criteria |
| Guoqing Wei et al. | Lee DW et al | CTCAE v5.0 | Lugano Treatment Response Criteria |
| Jia Wei et al. | Lee DW et al | CTCAE v4.03 | NCCN guidelines and Lugano Treatment Response Criteria |
| Jiaying Wu et al. | Lee DW et al | CTCAE v5.0 | NCCN guidelines and Lugano Treatment Response Criteria |
| ChenZeng et al. | Lee DW et al | CTCAE v5.0 | Lugano Treatment Response Criteria |
| Ying Zhang et al. | Lee DW et al | CTCAE v5.0 | Lugano Treatment Response Criteria |
| Yu Zhang et al. | Lee DW et al | NR | NCCN guidelines |
| Yeye Zhou et al. | Lee DW et al | CTCAE v4.03 | NR |

Abbreviations: CRS, cytokine release syndrome; ICANS, immune effector cell-associated neurotoxicity syndrome; CTCAE, the US National Cancer Institute Common Terminology Criteria for Adverse Events; NCCN, the US National Comprehensive Cancer Network; PET/CT, positron emission tomography/computed tomography scan; NR, no reported.

**Table A.4.** **Results of MINORS analysis for bias in individual studies**

| **Study** | **A clearly stated aim** | **Inclusion of consecutive patients** | **Prospective collection of data** | **Endpoints appropriate to the aim of the study** | **Unbiased assessment of the study endpoint** | **Follow-up period appropriate to the aim of the study** | **Loss to follow up less than 5%** | **Prospective calculation of the study size** | **Overall score** |
| --- | --- | --- | --- | --- | --- | --- | --- | --- | --- |
| Yang Cao et al. | ★★ | ★★ | ★★ | ★★ | ★★ | ★★ | ★★ | ★★ | 16 |
| Shaun Cordora et al. | ★★ | ★★ | ★★ | ★★ | ★★ | ★★ | ★★ | ★ | 15 |
| Hanren Dai et al. | ★★ | ★★ | ★★ | ★★ | ★★ | ★★ | ★★ | ★ | 15 |
| Yongxian Hu et al. | ★★ | ★★ | ★★ | ★★ | ★ | ★★ | ★★ | ★ | 14 |
| Shuangyou Liu et al. | ★★ | ★★ | ★★ | ★★ | ★★ | ★★ | ★★ | ★★ | 16 |
| Jay Y. Spiegel et al. | ★★ | ★★ | ★★ | ★★ | ★★ | ★★ | ★★ | ★★ | 16 |
| Na Wang et al. | ★★ | ★★ | ★★ | ★★ | ★★ | ★★ | ★★ | ★★ | 16 |
| Guoqing Wei et al. | ★★ | ★★ | ★★ | ★★ | ★★ | ★★ | ★★ | ★ | 15 |
| Jia Wei et al. | ★★ | ★★ | ★★ | ★★ | ★ | ★ | ★ | ★★ | 13 |
| Jiaying Wu et al. | ★★ | ★★ | ★★ | ★★ | ★★ | ★★ | ★★ | ★★ | 16 |
| ChenZeng et al. | ★★ | ★★ | ★★ | ★★ | ★★ | ★★ | ★ | ★ | 14 |
| Ying Zhang et al. | ★★ | ★★ | ★★ | ★★ | ★★ | ★★ | ★ | ★ | 14 |
| Yu Zhang et al. | ★★ | ★★ | ★★ | ★★ | ★★ | ★★ | ★★ | ★ | 15 |
| Yeye Zhou et al. | ★★ | ★★ | ★★ | ★★ | ★★ | ★★ | ★★ | ★ | 15 |

Abbreviations: MINORS, methodological index for non-randomized studies

**Table A.5.** **CD22 and CD19 dual-targeting chimeric antigen receptor T cell trials in patients with relapsed/refractory acute lymphoblastic leukemia in conference abstract**

| **ALL** | **PN** | **Patient age** | **Dual CAR targeting** | **Infused CAR T-cell dose** | **CAR construct and signaling/ generation** | **Origin type of CAR T cells** | **T cell culture time (days)** | **Transduction**  **method** | **Conditioning chemotherapy** | **OR** | **CR** | **MRD-/CR** | **CRS** | **sCRS** | **Neuro** | **Median follow-up (months)** |
| --- | --- | --- | --- | --- | --- | --- | --- | --- | --- | --- | --- | --- | --- | --- | --- | --- |
| Colleen Annesley et al, 2021 | 14 | NR | Cotransduction | 0.5 to 3 × 10^6^ SCRI-CAR19x22v2 CAR T cells/kg | scFv-4-1BB-CD3ζ (2nd generation) (anti-CD22 and anti-CD19) | Autologous | 7-12 | Lentivirus | Flu+Cyclo | 10/11 | 10/11 | 10/11 | 5/11 | 0 | 5/11 | NR |
| Wei Cui et al, 2021 | 47 | NR | Tandem | 1(0.5-2.5) x10^7^ CD19/CD22 CAR-T cells/kg | NR | NR | NR | NR | Flu+Cyclo | 47/47 | 47/47 | 40/47 | 41/47 | 8/47 | 1/47 | 21.83 |
| Noelle V Frey et al, 2021 | 13 | 46 (28-71) | Coadministration | 2.0x10^6^ CART22-65sf2 cells/kg and 2.0x10^6^ huCART19 cells/kg | NR | Autologous | NR | NR | Flu+Cyclo | 11/11 | 11/11 |  | 11/13 | 0 | 2/13 | 6.2 |
| Rebecca Alice Gardner et al, 2020 | 27 | NR | Cotransduction | 1 or 3x 10^6^ SCRI-CAR19x22v1 T cells/kg | scFv-4-1BB-CD3ζ (2nd generation) (anti-CD22 and anti-CD19) | Autologous | 7-11 | Lentivirus | Flu+Cyclo | 23/27 | 23/27 | 22/27 | 22/27 | 3/27 | 11/27 | NR |
| Pan J. et al, 2021 | 20 | 6 (1-15) | Coadministration | 10 (3.3 to 42.8) × 10^5^/kg CD19 CAR T cells and 10 (0.25 to 47.4) × 10^5^/kg CD22 CAR T cells | scFv-4-1BB-CD3ζ (2nd generation)(anti-CD19) and scFv-4-1BB-CD3ζ(2nd generation)(anti-CD22) | Autologous | 7-8 | Lentivirus | NR | 20/20 | 20/20 | 20/20 | 18/20 | NR | 4/20 | 27.3 |
| Liora M Schultz et al, 2019 | 12 | 23 (2-68) | Tandem | 1-3 x10^6^ CD19/CD22 bispecific CAR T cells/kg | scFv-4-1BB-CD3ζ (2nd generation) (anti-CD22 and anti-CD19) | Autologous | 7-11 | Lentivirus | Flu+Cyclo | 11/12 | 11/12 | NR | 9/12 | 1/12 | 2/12 | 9.5 |
| Haneen Shalabi et al, 2020 | 11 | 21 (5-28) | Tandem | 3-30 x 10^5^ bispecific CD19/CD22 CAR T-cell/kg | scFv-4-1BB-CD3ζ (2nd generation) (anti-CD22 and anti-CD19) | NR | NR | NR | NR | 8/11 | 4/11 | NR | 4/11 | 0 | 0 | NR |
| Junfang Yang et al, 2020 | 9 | 10 (3-48) | Tandem | 6.0×104/kg-2.25×10^5^/kg CD19/CD22 dual CAR-T (GC022F) | scFv-4-1BB-CD3ζ (2nd generation) (anti-CD22 and anti-CD19) | Autologous | 9-14 | Lentivirus | Flu+Cyclo | 9/9 | 9/9 | 8/9 | 6/9 | 0 | 1/9 | 3.3 |
| Junfang Yang et al, 2019 | 16 | 15 (5-45) | Tandem | 2.5-5×10^5^/kg-3-5×10^6^/kg CD19/CD22 dual CAR-T (GC022) | scFv-4-1BB-CD3ζ (2nd generation) (anti-CD22 and anti-CD19) | Autologous | 8-14 | Lentivirus | Flu+Cyclo | 8/11 | 8/11 | 6/11 | 16/17 | 0 | 0 | 2 |

Abbreviations: PN, patient number; OR, Overall response; CR, Complete response; MRD-/CR, Minimal residual disease negative/Complete response; CRS, Cytokine release syndrome; sCRS, severe cytokine release syndrome; Flu+Cyclo, Fludarabine+Cyclophosphamide; NR, not reported

**Table A.6.** **CD22 and CD19 dual-targeting chimeric antigen receptor T cell trials in patients with relapsed/refractory acute lymphoblastic leukemia in conference abstract**

| **NHL** | **PN** | **Disease type** | **Patient age** | **Combined therapy** | **Dual CAR targeting** | **Infused CAR T-cell dose** | **CAR construct and signaling/ generation** | **Origin type of CAR T cells** | **T cell culture time (days)** | **Transduction**  **method** | **Conditioning chemotherapy** | **OR** | **CR** | **CRS** | **sCRS** | **Neuro** |
| --- | --- | --- | --- | --- | --- | --- | --- | --- | --- | --- | --- | --- | --- | --- | --- | --- |
| E. Tholouli et al, 2020 | 23 | 17 DLBCL NOS, 6 tDLBCL | 57 (28-83) | anti-PD-1 antibody (Pembrolizumab) | Bicistronic vector | 50-450 x 10^6^ CD19/22 CAR T-cells | scFv-OX40-CD3 (2nd generation)(anti-CD19) and scFv-41BB-Cd3(2nd generation)(anti-CD22) | Autologous | 10-14 | Retrovirus | Flu+Cyclo | 11/16 | 9/16 | NR | 0 | 0 |
| Yang F. et al, 2021 | 7 | 6 DLBCL, 1 Burkitt | 43 (26-54) | Haplo-HSCT | Tandem | 3.6 (1.87-4.0) ×10^6^ allo-CD19/CD22 CAR-T/kg | NR | Allogenic | NR | NR | Busulfan, Fludarabine-based | 6/7 | 2/7 | 7/7 | 3/7 | 0 |
| Ying Zhang et al, 2021 | 11 | NR | NR | anti-PD-1 antibody | Coadministration | NR | CD19-4-1BB-CD3 z and CD22-4-1BB-CD3 (second-generation CAR-T cells) | NR | NR | Lentivirus | NR | 9/11 | 8/11 | 4/11 | 0 | 0 |

Abbreviations: PN, patient number; anti-PD-1, anti–programmed death-1; Haplo-HSCT, Haploidentical Haematopoietic stem cell transplantation; OR, Overall response; CR, Complete response; MRD-/CR, Minimal residual disease negative/Complete response; CRS, Cytokine release syndrome; sCRS, severe cytokine release syndrome; DLBCL NOS diffuse large B-cell lymphoma, not otherwise specified; tDLBCL, transformed diffuse large B-cell lymphoma; Flu+Cyclo, Fludarabine+Cyclophosphamide; NR, not reported

**Figure A.1. Forest plot showing partial remission in relapsed/refractory acute lymphoblastic leukemia (ALL) patients**


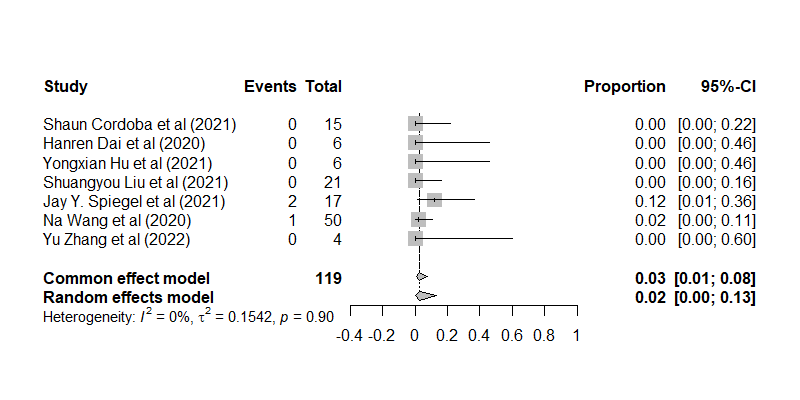


**Figure A.2. Forest plot showing minimal residual disease (MRD)-negative response in relapsed/refractory ALL patients**


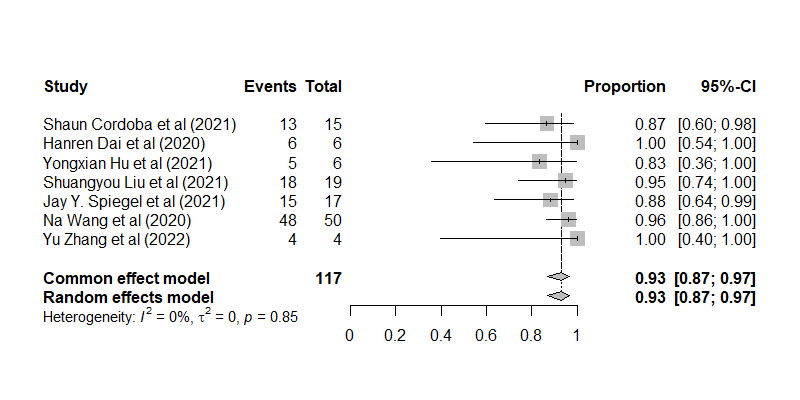


**Figure A.3. Forest plot for survival outcome in six months for ALL.** (A) Six-month overall survival. (B) Six-month event free survival.

**A**
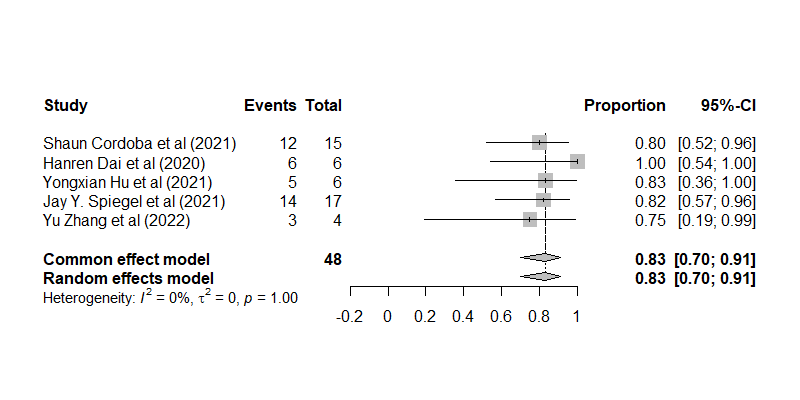
**B**
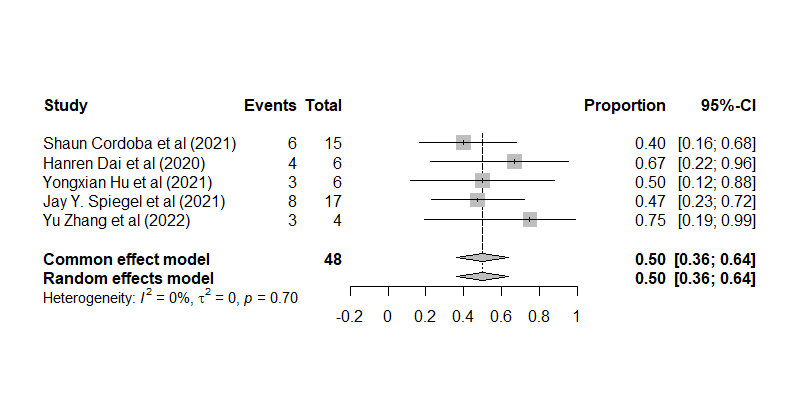


**Figure A.4. Forest plot for survival outcome in one year for ALL** (A) One-year overall survival. (B) One-year event free survival.

**A**


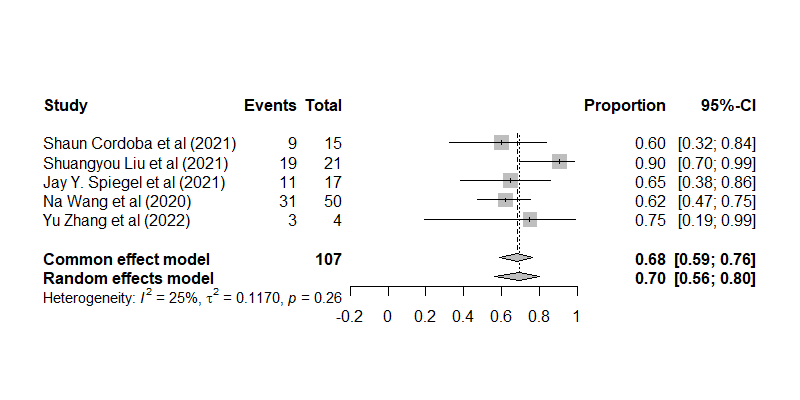


**B**


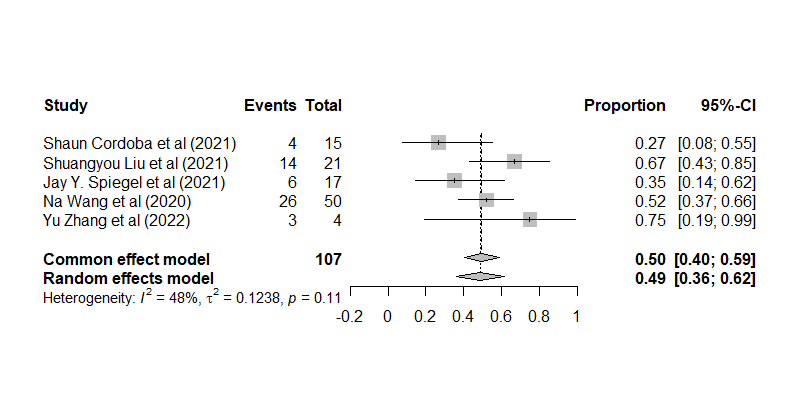


**Figure A.5. Forest plot showing partial remission in relapsed/refractory non-Hodgkin Lymphoma (NHL)**


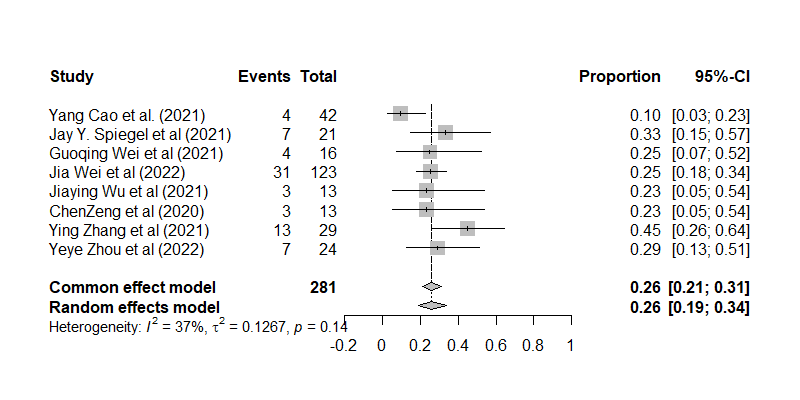


**Figure A.6. Subgroup analysis of complete remission in relapsed/refractory NHL** (A) Complete remission rates based on different dual-targeting approaches. (B) Complete remission rates based on CAR-T generation. (C) Complete remission rates based on combining with or without ASCT therapy. (D) Complete remission rates based on lymphodepletion pre-treatment.

**A**


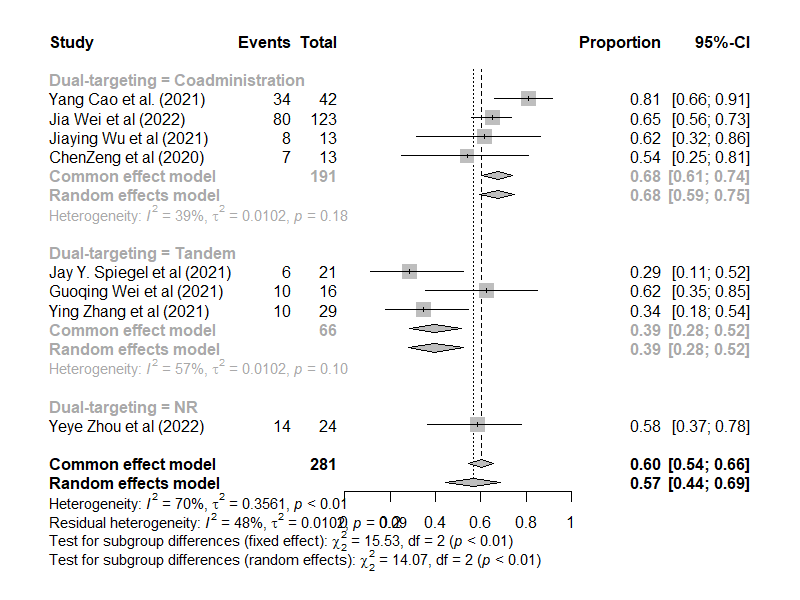


**B**


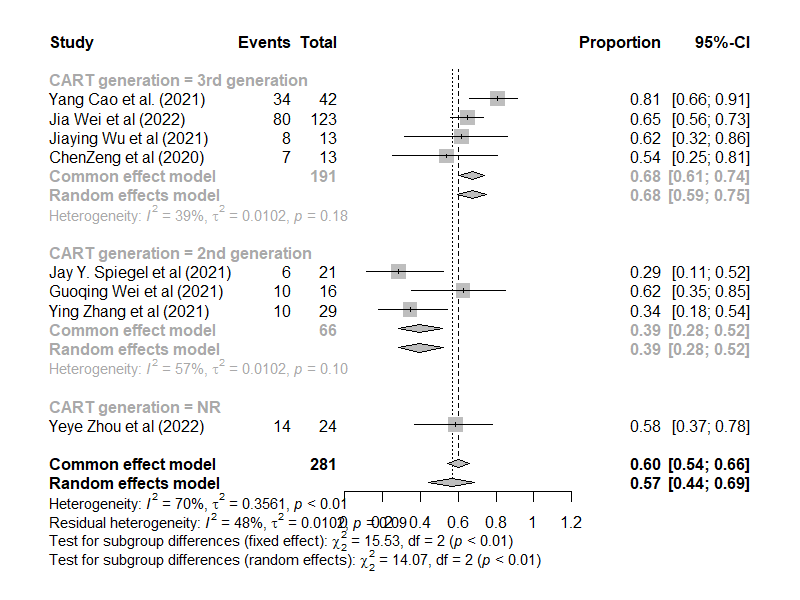


**C**
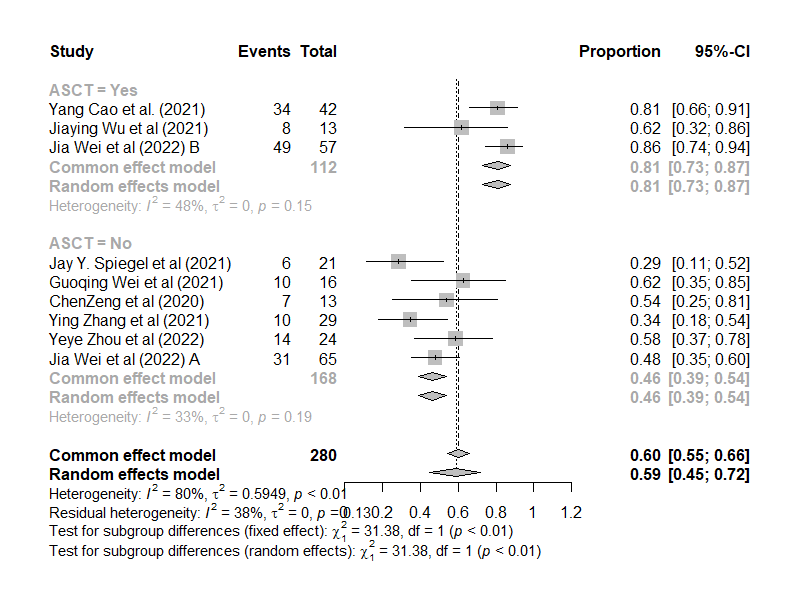


**D**


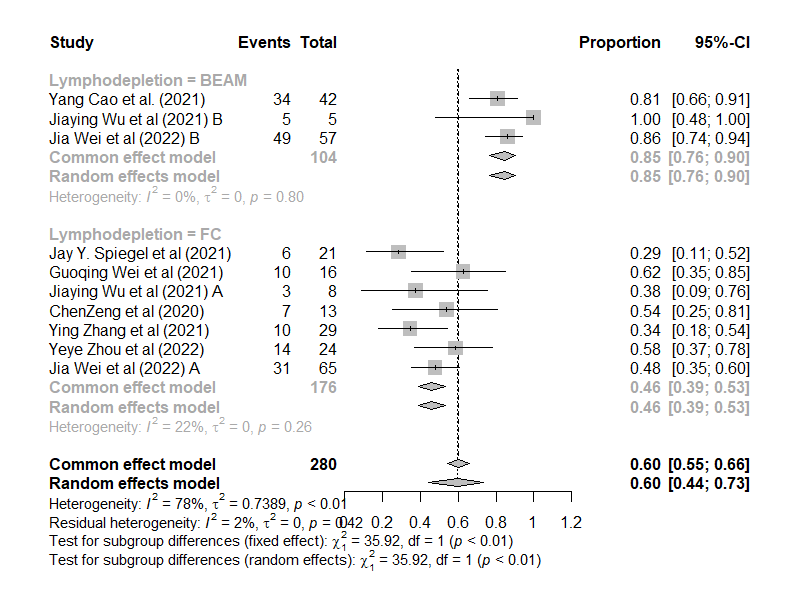


Abbreviations: NR, no reported; BEAM, carmustine, etoposide, cytarabine, and melphalan; FC, Fludarabine+Cyclophosphamide.

**Figure A.7. Forest plot for survival outcome in one year for NHL** (A) One-year overall survival. (B) One-year progression free survival.

**A**


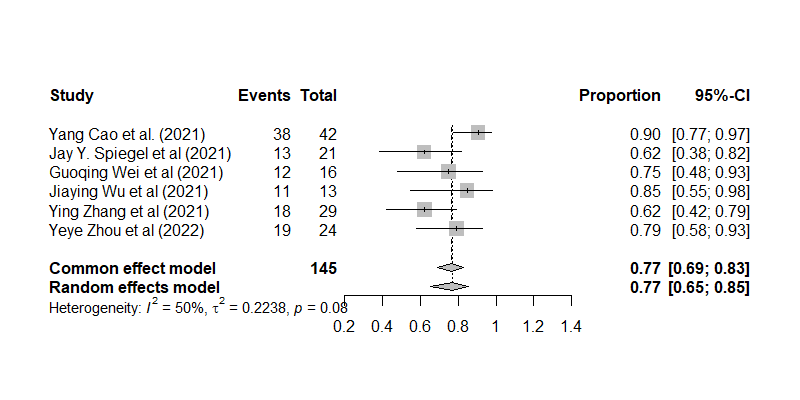


**B**


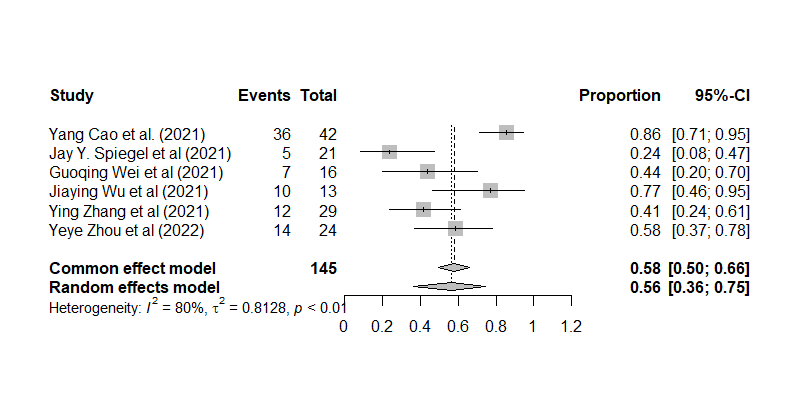


**Figure A.8. Subgroup analysis of Cytokine release syndrome (CRS) in relapsed/refractory ALL** (A) CRS rates based on different grading scale. (B) CRS rates based on CAR-T generation. (C) CRS rates based on dose of infused dual-targeting CAR-T cells.

**A**


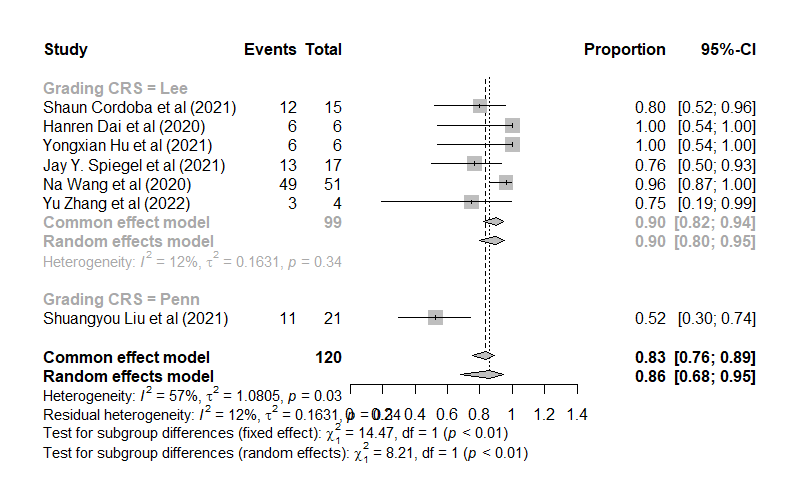


**B**


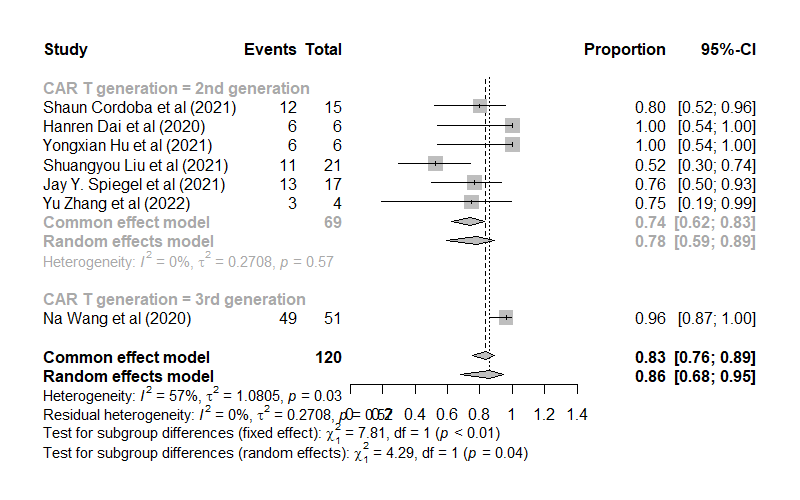


**C**
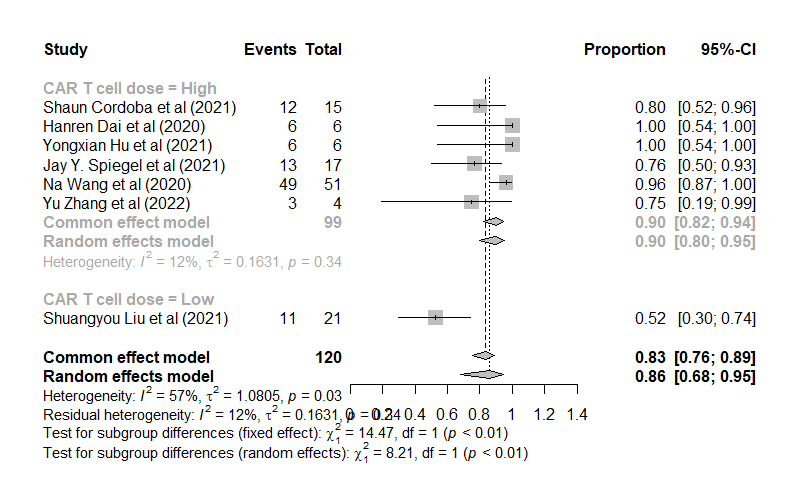


**Figure A.9. Subgroup analysis of Cytokine release syndrome in relapsed/refractory NHL** (A) CRS rates based on CAR-T generation. (B) CRS rates based on different dual-targeting approaches.

Abbreviations: NR, no reported

**A**


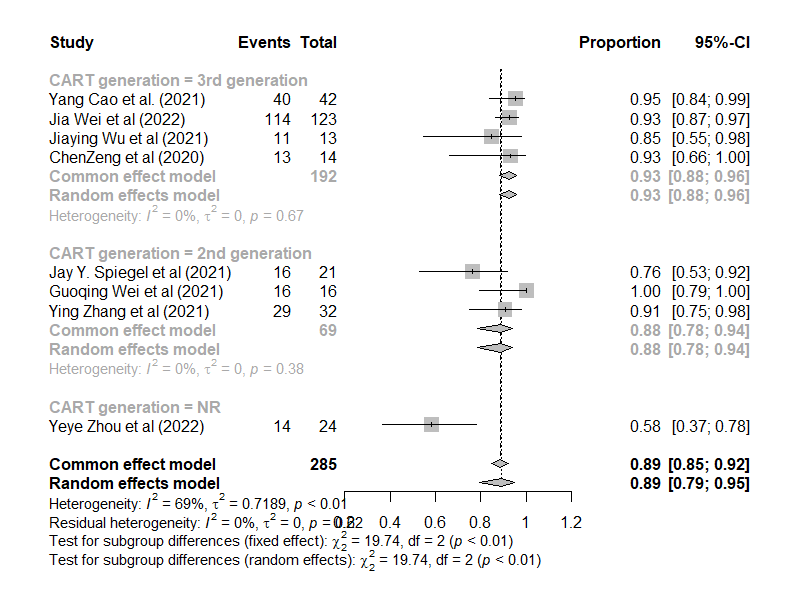


**B**


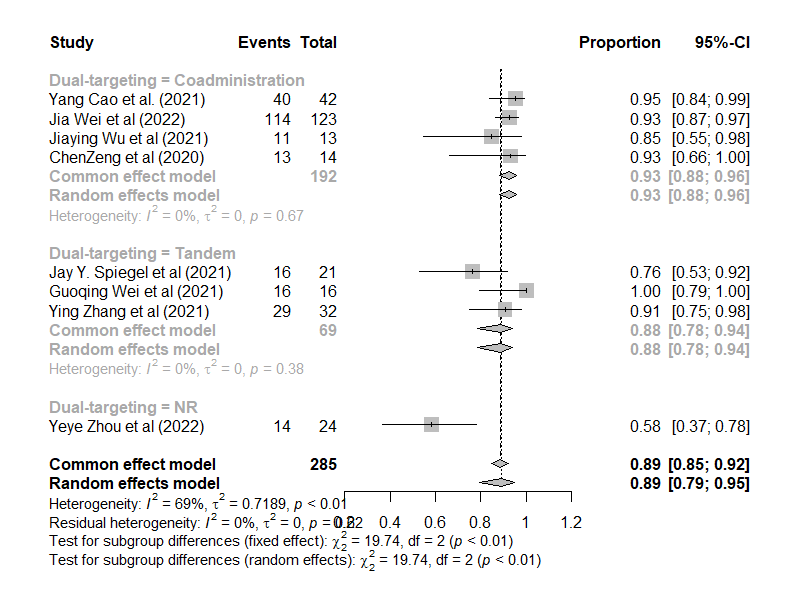


**Figure A.10 Funnel charts for the total overall response, complete remission, CRS and neurotoxicity, severe CRS in relapsed/refractory ALL and NHL patients**. (A) Funnel plot for overall response (B) Funnel plot for complete remission (C) Funnel plot for CRS (D) Funnel plot for neurotoxicity (E) Funnel plot for CRS in ALL trials (F) Funnel plot for overall response (G) Funnel plot for complete remission (G) Funnel plot for CRS (I) Funnel plot for neurotoxicity (J) Funnel plot for CRS in NHL trials.

**A**


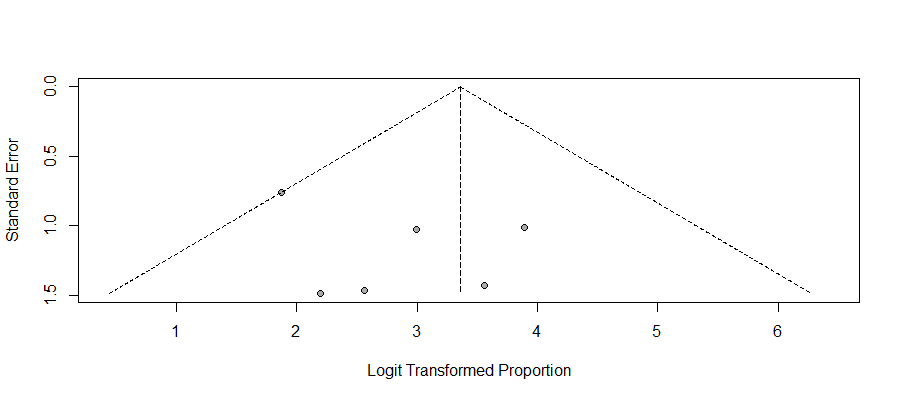


**B**


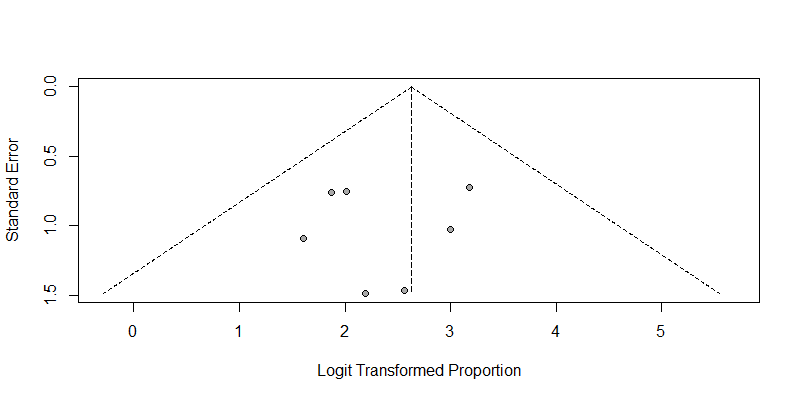


**C**


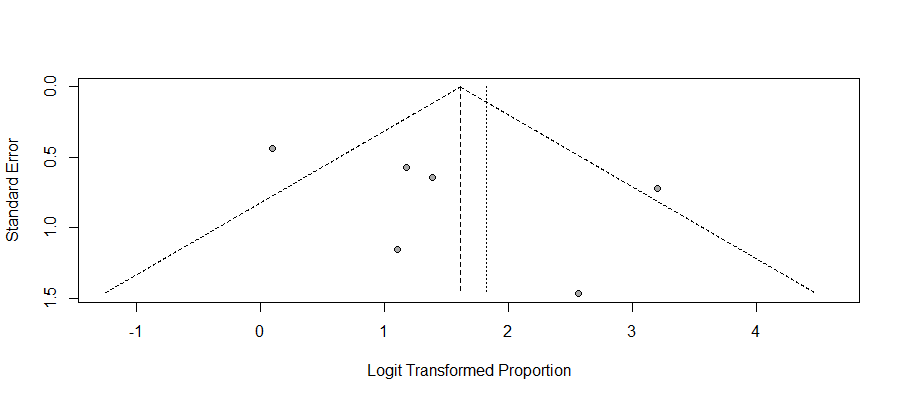


**D**


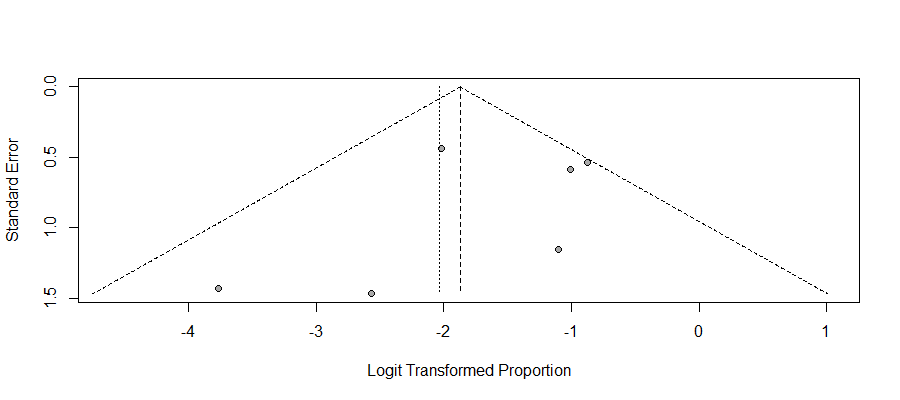


**E**


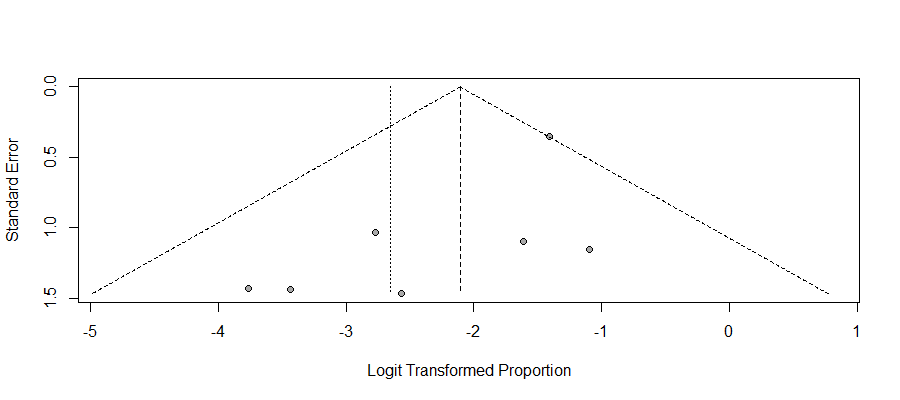


**F**


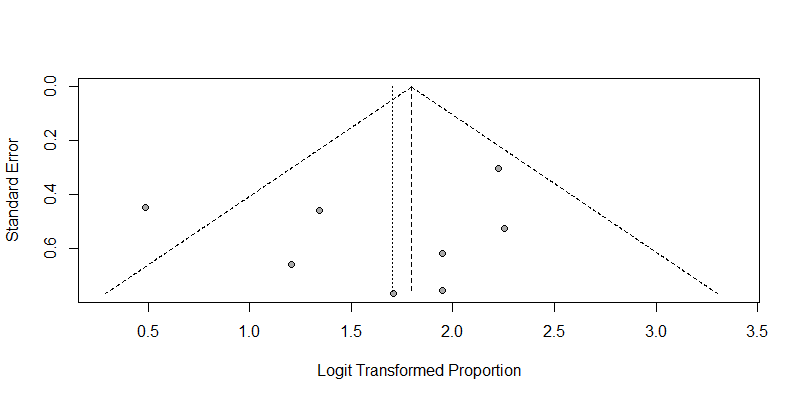


**G**


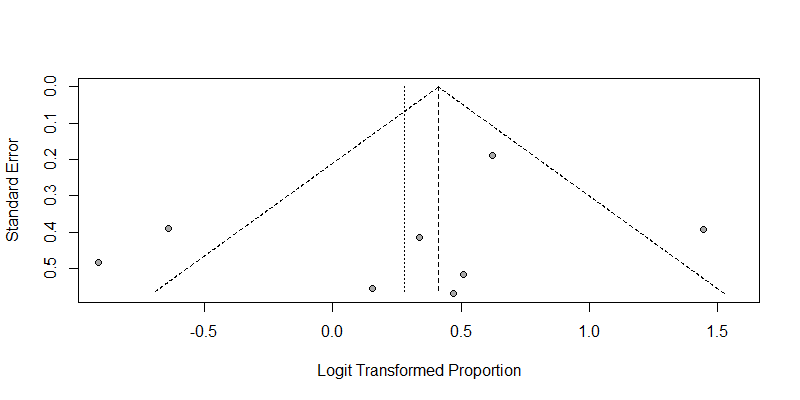


**H**


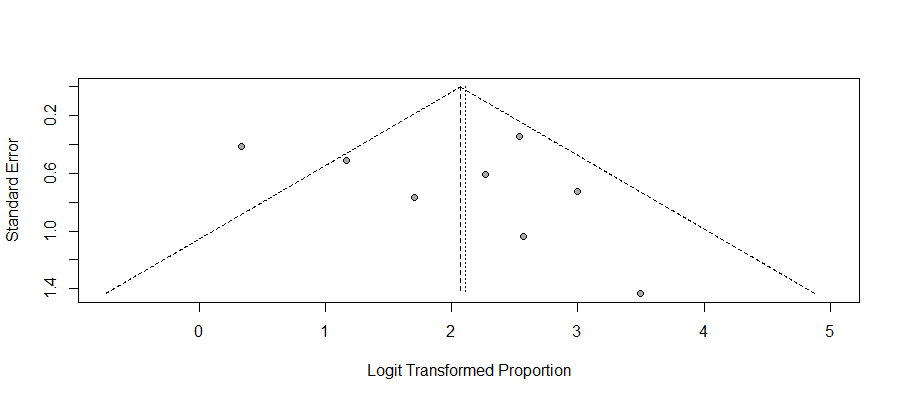


**I**


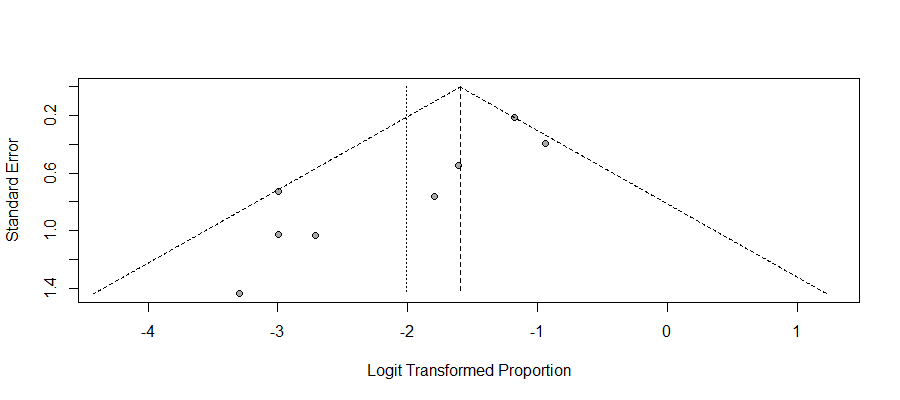


**J**
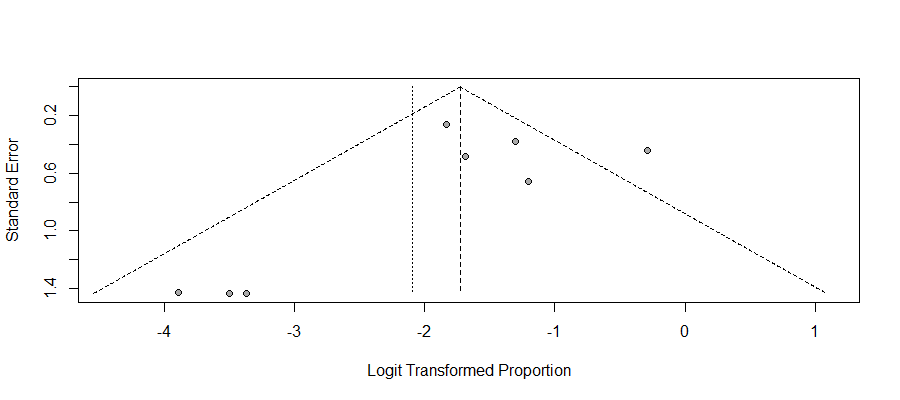

Supplement: Supplementary file 1 — Data S1. Supporting Information [file CAM4-12-18767-s001.docx]
